# Effects of exercise-based interventions on balance and functional mobility in adults with chronic stroke: a systematic review and meta-analysis

**DOI:** 10.3389/fpubh.2026.1930987

**Published:** 2026-10-13

**Authors:** Yating Zhan, Hao Bai, Tongtong Pan, Yue Li, Rong Wang

**Affiliations:** School of Nursing, University of South China, Hengyang, Hunan, China

**Keywords:** chronic stroke, exercise therapy, functional mobility, meta-analysis, physical therapy, postural balance, public health, rehabilitation

## Abstract

**Objective:**

To systematically evaluate, using the GRADE framework, the effects of exercise-based interventions on Berg Balance Scale, Timed Up and Go, and Functional Reach Test outcomes in adults with chronic stroke.

**Methods:**

We systematically searched PubMed, Embase, Web of Science, Scopus, and the Physiotherapy Evidence Database from inception to 29 June 2025 for randomized controlled trials investigating structured exercise-based interventions in adults with chronic stroke. Eligible studies enrolled adults at least 6 months after stroke onset and reported at least one validated balance or functional mobility outcome. Methodological quality was assessed using the Physiotherapy Evidence Database scale, and the certainty of evidence was rated using the GRADE approach. Meta-analyses were performed using random-effects models and expressed as mean differences (MDs) or standardized mean differences (SMDs) with 95% confidence intervals (CIs); exploratory univariable random-effects meta-regression was used to examine intervention duration and training frequency.

**Results:**

Sixty-nine randomized controlled trials involving 2,314 adults with chronic stroke were included. Exercise-based interventions improved Berg Balance Scale scores [MD = 2.54, 95% CI (1.83–3.26)], reduced Timed Up and Go test completion time [MD = −2.68 s, 95% CI (−3.72 to −1.65)], and improved Functional Reach Test performance [SMD = 0.65, 95% CI (0.25–1.05)]. The certainty of evidence was moderate for BBS, high for TUG, and moderate for FRT, with the degree of heterogeneity differing across outcomes.

**Conclusion:**

Exercise-based interventions were associated with improvements in BBS scores, TUG performance, and FRT outcomes in adults with chronic stroke, although the certainty and consistency of evidence varied across these measures. These findings support the potential role of exercise-based interventions in improving these selected outcomes. Larger pragmatic trials with standardized intervention reporting and longer follow-up are needed to clarify optimal training components, dose, delivery, and durability of effects.

**Systematic review registration:**

https://www.crd.york.ac.uk/PROSPERO/view/CRD42020156992, identifier CRD42020156992

## Introduction

Population aging and the growing burden of chronic non-communicable diseases have substantially increased the demand for long-term rehabilitation services. Rehabilitation is now recognized as an essential component of universal health coverage and a key strategy for improving functioning, reducing disability, and promoting health across the life course (1, 2). For patients living with chronic stroke, persistent impairments in balance and functional mobility not only reduce independence in activities of daily living but also increase the risk of falls, recurrent health-care utilization, and caregiving burden. Therefore, improving post-stroke balance and functional mobility is not only a clinical rehabilitation goal, but also an important public health issue related to disability prevention and long-term care.

Balance control is a key determinant of daily activities and an important predictor of walking ability and functional recovery after stroke (3). Balance impairments after stroke are common and may manifest as increased postural sway, asymmetric weight-bearing, reduced postural control, and impaired responses to external perturbations (4). These deficits can persist into the chronic phase, continuing to limit safe ambulation and community participation. Stroke rehabilitation guidelines emphasize the importance of balance, mobility, and task-specific training to improve functional recovery after stroke (5). The Berg Balance Scale (BBS) is one of the most widely used clinical measures for evaluating balance performance and fall-related risk in stroke populations (6). Functional mobility is another core outcome in chronic stroke rehabilitation. The Timed Up and Go (TUG) test assesses dynamic balance, gait performance, turning ability, and functional mobility and has demonstrated good reliability and clinical utility in patients with stroke (7).

Exercise-based interventions are widely recommended in stroke rehabilitation and encompass various modalities, including balance training, gait training, aerobic exercise, resistance training, task-oriented training, and technology-assisted exercise. Previous systematic reviews, Cochrane evidence, and clinical guidelines have shown that exercise-based interventions can improve motor function, physical fitness, and balance- and mobility-related outcomes after stroke (8–10). Current best-practice recommendations emphasize lower-extremity, balance, mobility, and aerobic training as key components of stroke rehabilitation, with training programs ideally being task-specific, progressive, intensive, and goal-oriented (11). A recent meta-analysis focusing on chronic stroke reported beneficial effects of exercise-based interventions on commonly used clinical measures, including BBS and TUG performance (12). Nevertheless, exercise-based interventions differ substantially in modality, duration, frequency, intensity, supervision, and training content. It therefore remains unclear whether the effects observed across commonly used balance and functional mobility measures vary according to intervention type and characteristics. Further synthesis focusing on commonly used clinical outcomes is therefore needed to clarify intervention-specific effects and support long-term rehabilitation planning.

Focusing on patients with chronic stroke is particularly important because spontaneous neurological recovery is most pronounced during the early months after stroke, whereas functional improvement tends to stabilize thereafter (3). Intervention effects observed in the chronic phase may therefore more directly reflect the contribution of the intervention itself. From a public health perspective, identifying effective exercise-based intervention strategies for chronic stroke survivors may help optimize rehabilitation resource allocation, support community-based functional maintenance, and reduce fall-related disability and long-term care needs (13).

Therefore, this systematic review and meta-analysis aimed to evaluate the effects of exercise-based interventions on selected balance and functional mobility outcomes in adults with chronic stroke, specifically the Berg Balance Scale, Timed Up and Go test, and Functional Reach Test. In addition, subgroup analyses were conducted to compare the effects of different exercise modalities and intervention characteristics. The findings may provide evidence to support individualized rehabilitation planning, community-based rehabilitation strategies, and long-term disability prevention for stroke survivors.

## Methods

### Study design and registration

This systematic review and meta-analysis were conducted and reported in accordance with the PRISMA 2020 statement and methodological guidance from the Cochrane Handbook. The review protocol was registered in PROSPERO (registration number: CRD42020156992). The PRISMA checklist is provided in Supplementary Table S1.

### Search strategy

Two reviewers independently searched PubMed, EMBASE, Web of Science, Scopus, and the Physiotherapy Evidence Database (PEDro) from database inception to 29 June 2025. The search strategy was developed according to the participants, intervention, comparator, outcome, and study design framework. It combined relevant subject headings and free-text terms related to stroke, chronic stroke, exercise therapy, physical rehabilitation, balance, functional mobility, and randomized controlled trials. The complete search strategies for each database are presented in Supplementary Table S2. Because PEDro has a different search interface, the PEDro search was documented using the search terms, filters, search date, and number of records retrieved. Reference lists of eligible studies and relevant reviews were also checked manually to identify additional studies.

### Eligibility criteria

Studies were selected according to the Population, Intervention, Comparator, Outcome, and Study design (PICOS) framework. The eligibility criteria were as follows: (1) Population: adults with chronic stroke, defined as at least 6 months after stroke onset, who had balance and/or functional mobility impairments; (2) Intervention: structured exercise-based interventions involving repeated physical exercise or task-specific physical practice; (3) Comparator: usual care, conventional rehabilitation, sham intervention, no intervention, waiting-list control, or another active rehabilitation intervention; (4) Outcomes: at least one validated measure of balance or functional mobility, including the Berg Balance Scale, Timed Up and Go test, Functional Reach Test, or another relevant measure; and (5) Study design: randomized controlled trials. Although the eligible interventions differed in modality, they all involved structured and repeated exercise or task-specific physical practice. For consistency, these interventions are referred to as “exercise-based interventions” throughout this review.

### Study selection

After duplicate records were removed, two reviewers independently screened the titles and abstracts of all retrieved records. The full texts of potentially eligible studies were then independently assessed by the same two reviewers according to the predefined eligibility criteria. Disagreements at either stage were resolved by consensus or consultation with a third reviewer. The study selection process, including the number of records identified, screened, excluded, and included, was documented using a PRISMA flow diagram.

### Data extraction

Two reviewers independently extracted data from all included studies using a standardized data extraction form. Extracted information included first author, publication year, country or region, study design, sample size, participant characteristics, mean age, post-stroke duration, intervention type, intervention frequency and duration, comparator characteristics, outcome measures, follow-up time points, and key findings. For quantitative synthesis, sample size, means, standard deviations, and relevant post-intervention or change-from-baseline values were extracted where available. Post-intervention values were preferentially used for the primary meta-analyses unless the available data required change scores. Any discrepancies were resolved through discussion with a third reviewer.

### Methodological quality assessment

All trials that met the PICOS and other eligibility criteria were assessed using the Physiotherapy Evidence Database (PEDro) scale (14). The scale consists of 11 criteria, of which 10 contribute to the total score, resulting in a score ranging from 0 to 10. PEDro scores were obtained from the PEDro database when available. For trials without an available PEDro database score, two reviewers independently evaluated methodological quality using the same PEDro scale; no alternative quality assessment instrument was used. Any disagreement was resolved through discussion or consultation with a third reviewer. Studies with PEDro scores of 6 or higher were classified as high quality, those with scores of 4–5 as moderate quality, and those with scores below 4 as low quality. A minimum PEDro score of 4 was required for inclusion in the final synthesis. This threshold was applied uniformly to exclude trials demonstrating very limited fulfillment of the scored methodological criteria, thereby reducing the potential influence of substantial methodological limitations on the pooled estimates. Detailed PEDro assessments of the included studies are presented in Supplementary Table S4.

### Statistical analysis

Meta-analyses were performed using Review Manager software, version 5.4.1, and Stata, version 18.0. Exploratory meta-regression analyses were conducted using R (version 4.5.1) with the metafor package. For continuous outcomes measured on the same scale, mean differences with 95% confidence intervals were calculated. For outcomes requiring standardization, standardized mean differences with 95% confidence intervals were calculated. When a study reported multiple eligible outcomes, data were extracted separately for each outcome-specific meta-analysis. Therefore, the number of participants differed across the BBS, TUG, and FRT analyses. For multi-arm trials, eligible intervention arms were combined when clinically appropriate, or shared control groups were split across comparisons to avoid double-counting. For the Berg Balance Scale and Functional Reach Test, positive effect estimates favored the intervention group. In contrast, negative effect estimates favored the intervention group for the Timed Up and Go test. Between-study heterogeneity was assessed using the I^2^ statistic, with values greater than 50% indicating moderate to substantial heterogeneity. Because the eligible interventions differed in training content, dose, delivery, and comparator conditions, random-effects models were used to estimate the average relative intervention effects across trials for the BBS, TUG, and FRT outcomes. The pooled estimates were therefore interpreted as average effects across the range of eligible exercise-based interventions and comparator conditions, rather than as the effect of a single standardized intervention against a uniform control condition.

Pre-specified subgroup analyses were performed by intervention type when at least three studies were available for pooling within each subgroup. In addition, *post hoc* exploratory subgroup analyses were conducted according to comparator condition to examine whether effect estimates differed according to the treatment received by the control group. Comparator conditions were classified as minimal/no additional intervention, usual/routine rehabilitation, or active comparator intervention, and between-subgroup differences were assessed using the test for subgroup differences. To further explore potential sources of heterogeneity, exploratory univariable random-effects meta-regression analyses were conducted to examine whether intervention duration (weeks) and training frequency (sessions/week), assessed separately, were associated with effect estimates for the BBS, TUG, and FRT. Other intervention characteristics, including training intensity and supervision approaches, were not quantitatively analyzed because of inconsistent definitions and insufficient standardized reporting across studies. Models were estimated using restricted maximum likelihood (REML) with Knapp–Hartung adjustment. Robustness was additionally examined for the BBS duration analysis by excluding the study with a condensed 10-consecutive-weekday schedule and for the BBS frequency analysis by excluding one study with an atypical daily home-practice schedule. Sensitivity analyses were conducted by sequentially omitting individual studies to assess the robustness of the pooled results (15). In addition, three-level random-effects meta-analyses were performed as sensitivity analyses to account for potential dependency among studies conducted within the same country. Effect sizes were nested within studies, and studies were nested within countries, with country specified as a higher-level clustering factor. Models were estimated using restricted maximum likelihood (REML). Publication bias was examined visually using funnel plots and statistically using Egger’s regression test when at least 10 studies were included in a meta-analysis. When evidence of publication bias was detected, the Duval and Tweedie trim-and-fill method was applied to estimate its potential influence on the pooled effect. Statistical significance was defined as a two-tailed *p*-value of less than 0.05.

### Certainty of evidence

The overall certainty of evidence for each outcome was assessed using the Grading of Recommendations Assessment, Development, and Evaluation approach. The certainty of evidence was rated as high, moderate, low, or very low based on risk of bias, inconsistency, indirectness, imprecision, and publication bias. Reasons for downgrading were documented for each outcome and are presented in the summary of findings table.

## Results

### Study selection

The initial database search identified 11,570 records. After removal of 11,024 duplicate records, 546 records were screened by title and abstract, of which 425 were excluded. The full texts of 121 articles were assessed for eligibility. Of these, 46 were excluded because of an ineligible study design (*n* = 7), insufficient data for extraction (*n* = 10), a post-stroke duration of less than 6 months (*n* = 29). The remaining 75 trials met the PICOS and other eligibility criteria and underwent PEDro assessment. Six trials with PEDro scores below 4 were subsequently excluded because they did not meet the minimum methodological quality threshold. Consequently, 69 randomized controlled trials (16–84) were included in the systematic review and meta-analysis. The study selection process and reasons for full-text exclusion are shown in the PRISMA flow diagram (Figure 1).

**Figure 1:**
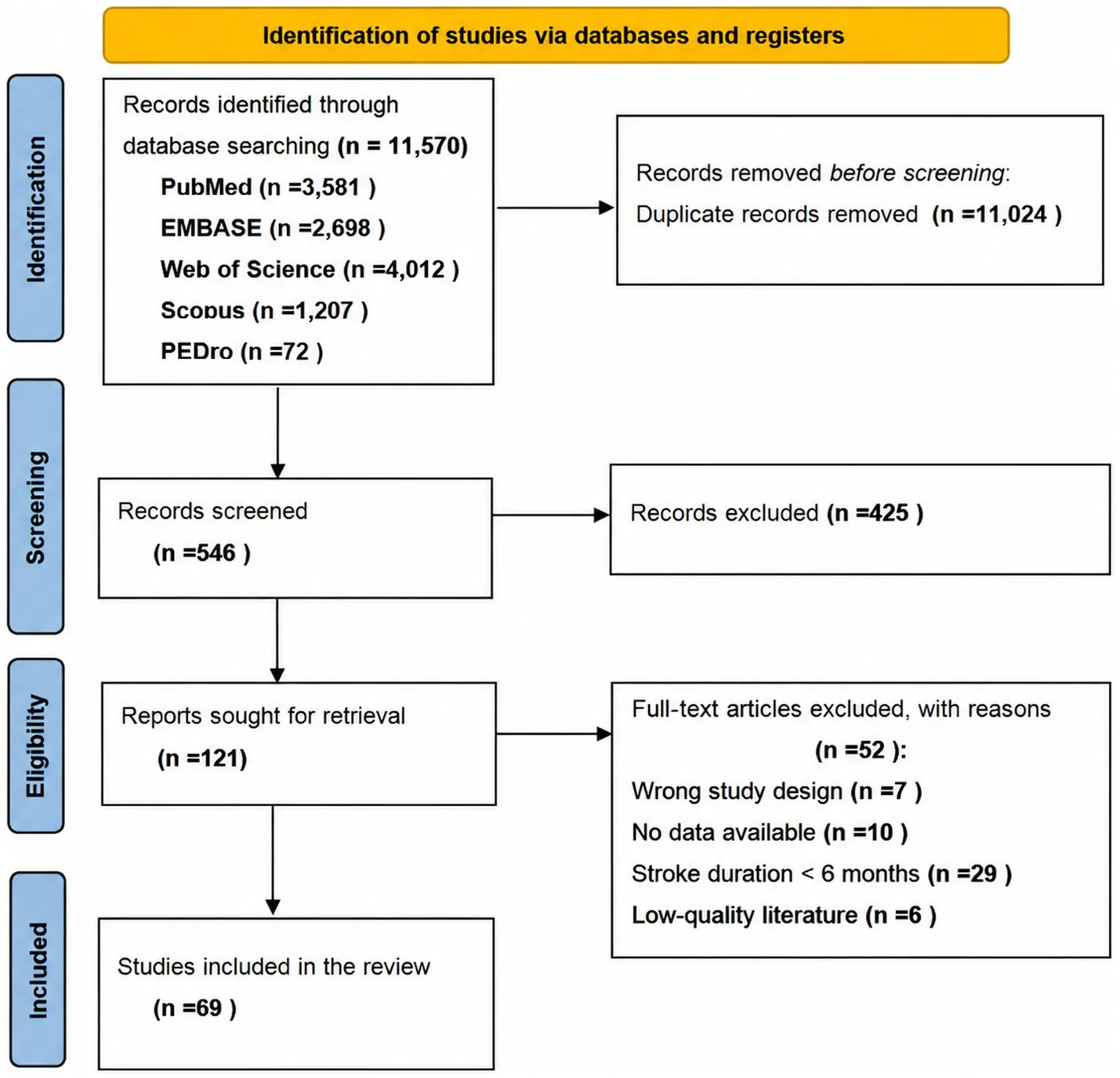
Flow diagram of literature search and study selection process.

### Study characteristics

The 69 included randomized controlled trials involved 2,314 participants from 16 countries (Table 1). All included studies focused on adults with chronic stroke or reported group-level stroke duration consistent with the predefined chronic stroke criterion of at least 6 months after stroke onset.

**Table 1:** Characteristics of the included studies.

Study	Country	Sample size, *n* (E/C)	Age, mean ± SD	Time since stroke	Setting	Intervention type	Control/comparator	Intervention duration	Outcomes
Pang 2005 (16)	Canada	60 (30/30)	E:65.8 ± 9.1; C:64.7 ± 8.4	≥1 year	Community	Multimodal rehabilitation	Active control	19 weeks	BBS
Kim 2013 (17)	South Korea	27 (9/9/9)	E1:55.3 ± 12.1; E2:54.8 ± 8.8; C:59.8 ± 8.9	>6 months post-stroke	Hospital	Balance training	Balance training	4 weeks	TUG; FRT
Quaney 2009 (18)	USA	38 (19/19)	E:64.1 ± 12.3; C:59.0 ± 14.7	≥6 months	Not reported	Aerobic + strengthening	Stretching	8 weeks	BBS
Lee 2014 (19)	South Korea	21 (10/11)	E:47.9 ± 12.0; C:54.0 ± 11.9	≥6 months	Not reported	VR + balance training	Conventional rehabilitation	4 weeks	BBS, TUG
Bayouk 2006 (20)	Canada	16 (8/8)	E:68.4 ± 7.1; C:62.0 ± 4.6	≥6 months	Community	Balance training	Active control	8 weeks	CoP
Middleton 2014 (21)	USA	38 (19/19)	E:61.4 ± 15.7; C:60.7 ± 11.4	>6 months	Not reported	Gait training	Overground gait	10 consecutive weekdays	BBS
Dias 2007 (22)	Portugal	40 (20/20)	E:70.4 ± 7; C:68.0 ± 11	>12 months	Not reported	Gait training	Conventional rehabilitation	5 weeks	COP
Wang 2015 (23)	China	51 (25/26)	E:62.0 ± 9.5; C:65.4 ± 10.6	>6 months	Community	Other	No intervention	12 weeks	BBS
Globas 2012 (24)	Germany	36 (18/18)	E:68.6 ± 6.7; C:68.7 ± 6.1	>6 months	Community	Gait + aerobic training	Conventional rehabilitation	13 weeks	BBS
Pandian 2014 (25)	India	39 (20/19)	E:44.5 ± 13.6; C:40.2 ± 15.0	E: 13 months; C: 20 months	Community	Strengthening	Conventional rehabilitation	8 weeks	BBS, TUG, FRT
Farqalit 2013 (26)	India	40 (20/20)	E:58.7 ± 4.2; C:60.1 ± 4.8	>6 months	Not reported	Balance + gait + strengthening	Stretching	4 weeks	BBS, TUG
Choi 2015 (27)	South Korea	37 (19/18)	E:49.1 ± 11.9; C: 49.3 ± 8.3	≥6 months	Hospital	Dual-task gait training	Treadmill	4 weeks	TUG
Cho 2014 (28)	South Korea	30 (15/15)	E:65.9 ± 5.7; C:63.5 ± 5.5	>6 months	Not reported	Gait training	Conventional rehabilitation	6 weeks	BBS,TUG
Tankisheva 2014 (29)	Belgium	13 (6/7)	E:57.4 ± 13; C:65.3 ± 3.7	E: 7.71 ± 8.6 years; C: 5.28 ± 3.6 years	Not reported	Balance + vibration	No intervention	6 weeks	SOT
Cho 2013 (30)	South Korea	28 (15/13)	E:53.9 ± 12.6; C:53.9 ± 12.4	>6 months post-stroke	Hospital	Gait + motor imagery	Treadmill	6 weeks	TUG; FRT
Kang 2012 (31)	South Korea	30 (10/10/10)	E1:55.9 ± 6.5; E2:56.3 ± 7.6; C:56.1 ± 7.8	E1: 14.1 ± 4.4 months; E2: 13.5 ± 4.0 months; C: 15.1 ± 7.4 months	Not reported	Gait training	Conventional rehabilitation	4 weeks	TUG; FRT
Marin 2013 (32)	Spain	20 (11/9)	E:62.3 ± 10.6; C:64.4 ± 7.6	E: 4.3 ± 2.0 years; C: 4.3 ± 3.0 years	Not reported	Balance training	Active control	12 weeks	BBS
Gok 2008 (33)	Turkey	30 (15/15)	E:55.1 ± 11.4; C:59.7 ± 4.8	E: 545.2 months; C: 99.9 months	Hospital	Balance training	Conventional rehabilitation	4 weeks	FM
Marigold 2005 (34)	Canada	48 (22/26)	E:68.1 ± 9.0; C:67.5 ± 7.2	≥12 months	Community	Balance + sensory training	Stretching	10 weeks	BBS, TUG
Lee 2012 (35)	South Korea	40 (20/20)	E:53.8 ± 11.3; C:54.1 ± 11.1	E: 13.30 ± 5.89 months; C: 14.00 ± 6.34 months	Not reported	Balance training	Conventional rehabilitation	4 weeks	BBS; TUG
Yen 2008 (36)	China	14 (7/7)	E:57.3 ± 16.4; C:56.1 ± 12.7	E: 1.97 ± 0.61 years; C: 1.96 ± 2.42 years	Not reported	Gait training	Stretching	4 weeks	BBS
Llorens 2015 (37)	Spain	20 (10/10)	E:58.3 ± 11.6; C:55.0 ± 11.6	E: 587.6 ± 222.1 days; C: 407.5 ± 232.4 days	Community	VR training	Conventional rehabilitation	4 weeks	BBS
Yang 2011 (38)	China	14 (7/7)	E:56.3 ± 10.2; C:65.7 ± 5.9	≥6 months	Community	Other	Treadmill	3 weeks	CoP
Jin 2012 (39)	China	136 (68/68)	E:57 ± 6; C:56 ± 7	>6 months	Hospital	Aerobic training	Overground gait	8 weeks	BBS
Cha 2014 (40)	South Korea	20 (10/10)	E:59.8 ± 11.7; C:63.0 ± 14.1	E: 14.5 ± 5.5 months; C: 14.7 ± 5.4 months	Not reported	Gait training	Conventional rehabilitation	6 weeks	BBS
Brogardh 2012 (41)	Sweden	31 (16/15)	E:61.3 ± 8.5; C:63.9 ± 5.8	E: 37.4 ± 31.8 months; C: 33.1 ± 29.2 months	Not reported	Whole-body vibration	Active control	6 weeks	BBS
Westlake 2009 (42)	USA	16 (8/8)	E:58.6 ± 16.9; C:55.1 ± 13.6	E: 43.8 months; C: 26.8 months	Not reported	Robot-assisted gait training	Treadmill	4 weeks	BBS
Schmid 2012 (43)	USA	39 (29/10)	E:63.9 ± 8.7; C:60.2 ± 8.9	≥6 months	Not reported	Balance + mind-body exercise	No intervention	8 weeks	BBS
Hung 2014 (44)	China	28 (13/15)	E:55.4 ± 10.0; C:53.4 ± 10.0	≥6 months	Community	Multimodal rehabilitation	Conventional rehabilitation	12 weeks	TUG, FRT
Immink 2014 (45)	Australia	22 (11/11)	E:56 ± 13.6; C:63 ± 17.4	≥9 months	Not reported	Mind-body + sensory training	No intervention	10 weeks	BBS
Bonan 2004 (46)	France	20 (10/10)	E:49.5 ± 10; C:49 ± 17	>12 months	Community	Sensory training	Active control	4 weeks	SOT
Page 2008 (47)	USA	7 (4/3)	E+C: 61.3 ± 12.3	>1 year post-stroke	Community	Multimodal rehabilitation	Home exercise	8 weeks	BBS
Wu 2014 (48)	USA	28 (14/14)	E:53.6 ± 8.9.; C:57.4 ± 9.8	>6 months	Not reported	Gait + strengthening	Treadmill	6 weeks	BBS
Au-Yeung 2009 (49)	China	108 (56/52)	E:61.7 ± 10.7; C:65.9 ± 10.5	>6 months	Community	Mind-body exercise	Stretching	12 weeks	TUG
Noh 2008 (50)	South Korea	20 (10/10)	E:61.9 ± 10.1; C:66 ± 11.4	E: 2.8 ± 3.8 years; C: 1.6 ± 1.7 years	Community	Aquatic + mind-body exercise	Active control	8 weeks	BBS
Rydwik 2006 (51)	Sweden	17 (8/9)	E:74.9 ± 8.7; C:75.3 ± 4.9	Overall: 48.7 (19.6) months; E: 42.6 (18.2) months; C: 54.9 (20) months	Community	Other	No intervention	6 weeks	Romberg
Peurala 2005 (52)	Finland	45 (15/15/15)	E1: 53.3 ± 8.9; E2: 51.2 ± 7.9; C:52.3 ± 6.8	>6 months	Hospital	Gait training	Overground gait	3 weeks	CoP
Chen 2014 (53)	China	30 (15/15)	E:53.7 ± 11.1; C:54.8 ± 8.1	2.6 ± 1.9 years	Not reported	Gait training	Conventional rehabilitation	4 weeks	BBS
Kim 2009 (54)	South Korea	24 (12/12)	E:52.4 ± 10.1; C:51.8 ± 7.1	E: 25.91 ± 9.96 months; C: 24.25 ± 8.87 months	Not reported	VR + balance training	Conventional rehabilitation	4 weeks	BBS
Cho 2012 (55)	South Korea	22 (11/11)	E:65.3 ± 8.4; C:63.1 ± 6.9	≥6 months	Not reported	VR + balance training	Conventional rehabilitation	6 weeks	BBS, TUG
Chu 2004 (56)	Canada	12 (7/5)	E:61.9 ± 9.4; C:63.4 ± 8.4	E: 3.0 ± 2.0 years; C: 4.2 ± 2.1 years	Community	Aquatic + aerobic training	Active control	8 weeks	BBS
Lau 2012 (57)	China	82 (41/41)	E:57.3 ± 11.3; C:57.4 ± 11.1	>6 months	Community	Balance + vibration	Active control	8 weeks	BBS
Yuen 2021 (58)	China	58 (29/29)	E:63.1 ± 10.6; C:62.0 ± 13.1	E: 23.1 ± 21.5 months; C: 25.3 ± 21.6 months	Community	Balance + mind-body exercise	Stretching	16 weeks	TUG
Yang 2020 (59)	South Korea	30 (15/15)	E:61.53 ± 9.24; C:63.33 ± 9.43	E: 16.67 ± 5.38 months; C: 17.33 ± 11.91 months	Rehabilitation outpatient center	Balance training	Sham control	4 weeks	TUG; FRT
Saleh 2019 (60)	Egypt	50 (25/25)	E:49.53 ± 1.8; C:50 ± 1.96	6 months to 1 year	Community	Aquatic + dual-task training	Active control	6 weeks	Biodex
Saha 2021 (61)	India	30 (15/15)	E:52.07 ± 3.6736; C:52.40 ± 3.906	≥6 months	Community	Gait training	Conventional rehabilitation	4 weeks	BBS; TUG
Suh 2014 (62)	South Korea	16 (8/8)	E: 61.00 ± 14.48; C:70.63 ± 12.42	E: 386.38 ± 283.22 days; C: 224.25 ± 213.03 days	Hospital	Gait training	Active control	3 weeks	stability index
Yu 2020 (63)	China	71 (35/36)	E:63.03 ± 8.92; C:58.69 ± 9.72	>10 years	Hospital	Mind-body exercise	Conventional rehabilitation	12 weeks	BBS
Hong 2020 (64)	South Korea	17 (8/9)	E: 56.63 ± 8.78; C:66.22 ± 11.55	≥6 months	Hospital	Balance + dual-task training	Balance training	4 weeks	BBS; TUG
Kim 2015 (65)	South Korea	17 (10/7)	Not reported	≥6 months	Hospital	VR + gait training	Conventional rehabilitation	4 weeks	APSS; PSPL
Lee 2018 (66)	South Korea	20 (10/10)	E:59.80 ± 6.92; C:60.20 ± 8.24	>6 months	Hospital	Multimodal rehabilitation	Active control	6 weeks	BBS; TUG
Henrique 2019 (67)	Brazil	31 (16/15)	E:76.19 ± 10.09; C:76.20 ± 10.41	>6 months	Community	Multimodal rehabilitation	Active control	12 weeks	BBS
Kwag 2025 (68)	South Korea	20 (10/10)	E:50.6 ± 9.2; C:58.9 ± 6.8	≥6 months	Hospital	Multimodal rehabilitation	Active control	4 weeks	TUG
Choi 2016 (69)	South Korea	36 (12/12/12)	E:61.25 ± 5.59 C1:62.58 ± 5.51 C2:61.92 ± 6.08	>6 months	Hospital	VR training	Active control	4 weeks	TUG; FRT
Zhao 2022 (70)	China	40 (20/20)	E:60.4 ± 12.32; C:54.45 ± 13.94	E: 20.14 months; C: 8 months	Hospital	Multimodal rehabilitation	Active control	4 weeks	BBS, TUG
Lee 2015 (71)	South Korea	36 (18/18)	Not reported	>6 months	Hospital	Motor imagery + sensory training	Active control	8 weeks	TUG
Bang 2016 (72)	South Korea	18 (9/9)	E:53.56 ± 3.94; C:53.67 ± 2.83	E: 11.56 ± 2.60 months; C: 12.56 ± 2.65 months	Hospital	Robot-assisted gait training	Treadmill	4 weeks	BBS
Lee 2016 (73)	South Korea	30 (15/15)	E:53.9 ± 9.59; C:57.2 ± 14.57	≥6 months	Community	Motor imagery	Active control	6 weeks	BBS; TUG
Yong-Wook 2015 (74)	South Korea	37 (18/19)	E:53.4 ± 12.1; C:51.8 ± 13.7	E1: 32.4 ± 21.7 months; E2: 31.4 ± 19.2 months; C: 165.2 ± 8.7 months	Hospital	Gait training	Treadmill	4 weeks	COP
Hyung Young Lee 2015 (75)	South Korea	24 (12/12)	E:45.91 ± 12.28; C:49.16 ± 12.85	>6 months	Hospital	VR + balance training	Conventional rehabilitation	6 weeks	FRT
Korkusuz 2025 (76)	Turkey	25 (13/12)	E:61.0 ± 4.88; C:62.00 ± 6.63	E: 19.23 ± 4.91 months; C: 18.67 ± 5.67 months	Rehabilitation outpatient center	Multimodal rehabilitation	Conventional rehabilitation	6 weeks	TUG; FRT
Zhu 2016 (77)	China	28 (14/14)	E:56.6 ± 6.9; C:57.1 ± 8.6	>6 months post-stroke	Rehabilitation outpatient center	Aquatic therapy	Conventional rehabilitation	4 weeks	BBS; FRT
Junata 2021 (78)	China	30 (16/14)	E:60.6 ± 5.5; C:60.1 ± 5.8	>1 year	Community	Other	Conventional rehabilitation	7 weeks	BBS; TUG
Chang 2021 (79)	China	16 (8/8)	E:52.39 ± 6.06; C:54.38 ± 14.05	≥6 months	Hospital	Gait training	Active control	4 weeks	BBS; TUG
Mustafaoğlu 2018 (80)	Turkey	45 (15/15/15)	E1:52.8 ± 13.8; E2:53.7 ± 11.6; C:52.6 ± 14.7	E1: 12.5 months (range 3-36); E2: 12.0 months (range 7-18); C: 11.0 months (range 4-28)	Hospital	Robot-assisted gait training	Conventional rehabilitation	6 weeks	BBS; TUG
Cui 2024 (81)	South Korea	30 (10/10/10)	E1:52.60 ± 17.83; E2: 51.60 ± 9.63; C: 53.00 ± 12.77	>6 months	Hospital	Balance training	Balance training	4 weeks	BBS; TUG
Park 2021 (82)	South Korea	52 (15/18/19)	E1:58.07 ± 7.14; E2:58.11 ± 7.15; C:60.79 ± 6.75	E1: 8.73 ± 4.99 months; E2: 13.50 ± 6.81 months; C: 13.16 ± 6.43 months	Hospital	Balance training	Conventional rehabilitation	4 weeks	TUG
Çoban 2024 (83)	Turkey	30 (15/15)	E:64.5 ± 11.3; C:64.2 ± 11.8	E: 20.3 ± 21.01 months; C: 18.93 ± 21.07 months	Hospital	Multimodal rehabilitation	Conventional rehabilitation	4 weeks	BBS; TUG
Park 2018 (84)	South Korea	40 (12/12/16)	E1:55.58 ± 10.42; E2:56.66 ± 4.39; C:57.50 ± 9.90	>3 years	Hospital	VR + robot-assisted gait training	Active control	6 weeks	BBS; TUG; 10MWT

Values are presented as mean ± SD unless otherwise stated. E, experimental group; C, control group; VR, virtual reality; BBS, Berg Balance Scale; FRT, Functional Reach Test; CoP, center of pressure; TUG, Timed Up and Go test. Intervention type and control/comparator were standardized for readability.

Intervention protocols typically lasted 30–60 min per session, were delivered 3–5 times per week, and continued for 4–8 weeks in most studies. The weekly training dose was approximately 90–300 min, and the total intervention duration ranged from 3 to 18 weeks. Most interventions were therapist-supervised and delivered in structured rehabilitation settings, whereas a few studies incorporated home-based or partially supervised exercise programs. However, intervention intensity, progression criteria, and adherence reporting varied across studies.

The interventions covered a broad range of exercise-based approaches, including balance and functional weight-shifting training, gait training, multisensory or sensory input interventions, aerobic or strengthening-based exercise, technology-assisted training, mind–body exercise, dual-task training, and other multimodal exercise programs. Control conditions varied across trials and included usual care, conventional rehabilitation, no intervention or waiting-list control, sham intervention, and active rehabilitation comparators. Accordingly, the included comparisons did not represent a single uniform intervention–control contrast, and comparator intensity ranged from no additional treatment to active rehabilitation. For subgroup analyses, interventions were categorized by primary therapeutic objective into five groups: balance or functional weight-shifting training, gait training, multisensory training, high-intensity aerobic training, and other exercise modalities. Studies excluded after full-text assessment are summarized in Supplementary Table S3.

### Quality appraisal

All 75 trials that met the PICOS and other eligibility criteria were assessed using the PEDro scale. Six trials scored below 4 and were excluded from the final synthesis because they did not meet the minimum methodological quality threshold. The remaining 69 randomized controlled trials had PEDro scores ranging from 4 to 9 out of 10. Of these, 50 trials were classified as high quality and 19 as moderate quality. The detailed methodological quality assessments of the included trials are presented in Supplementary Table S4.

### Certainty of evidence

The certainty of evidence for the pooled outcomes was assessed using the GRADE approach (Supplementary Table S5). Evidence from randomized controlled trials was initially rated as high certainty and was downgraded when serious concerns were identified regarding risk of bias, inconsistency, indirectness, imprecision, or publication bias. The certainty of evidence was rated as moderate for BBS because potential small-study effects were detected, and high for TUG, because no serious concerns were identified across the GRADE domains, and moderate for FRT because of inconsistency related to moderate heterogeneity.

### Meta-analysis

#### Berg Balance Scale (BBS)

Forty-three studies involving 1,460 participants were included in the BBS analysis. Exercise-based interventions significantly improved BBS scores compared with control interventions (MD = 2.54, 95% CI: 1.83 to 3.26, *p* < 0.00001, I^2^ = 49%) (Figure 2).

**Figure 2:**
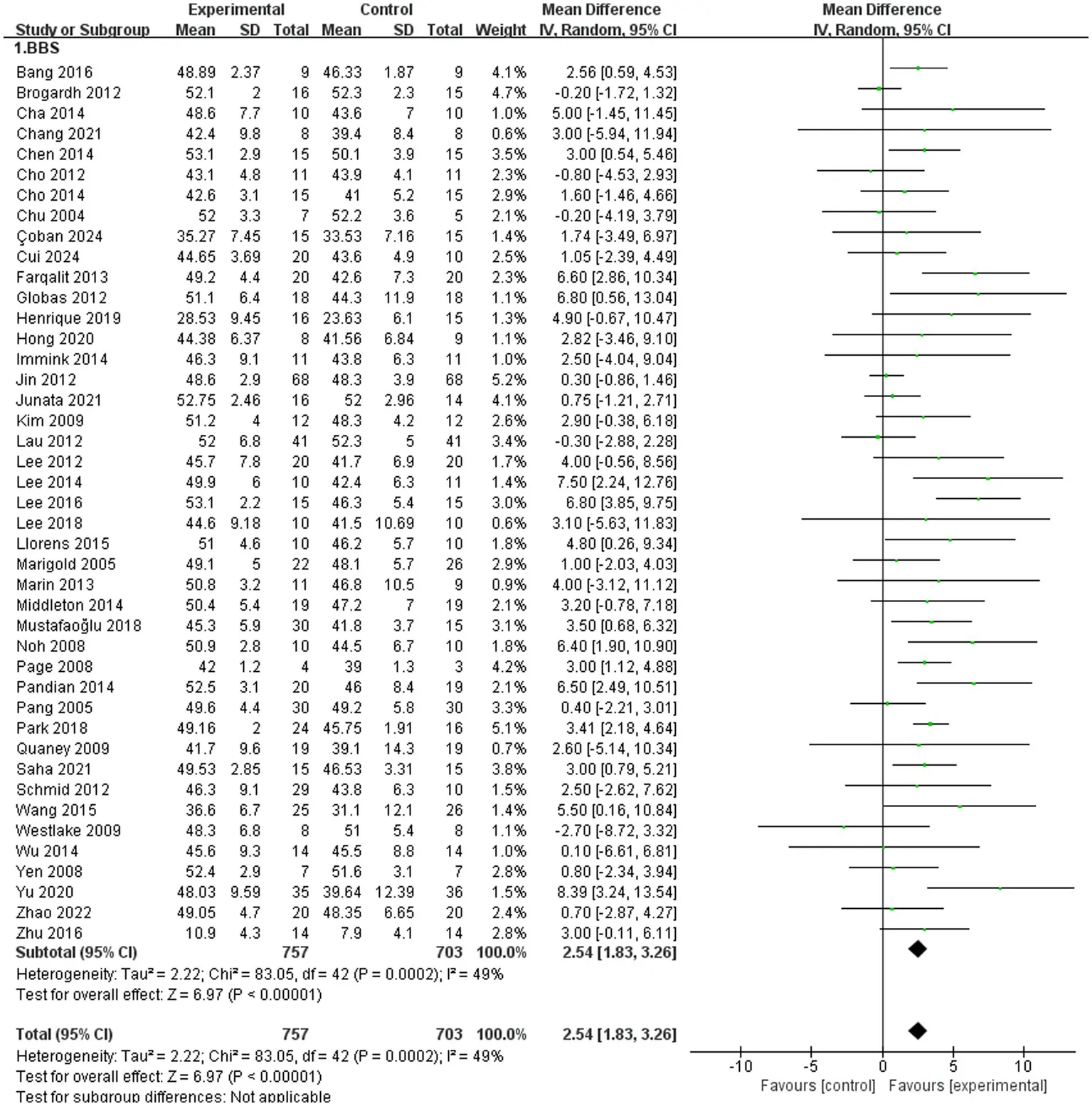
Forest plot showing the effects of exercise-based interventions on BBS scores in patients with chronic stroke. The Berg Balance Scale (BBS) was used as the outcome measure. Data are presented as mean difference (MD) with 95% confidence intervals (CIs), calculated using a random-effects model.

#### Timed Up and Go (TUG)

Thirty-one studies involving 1,083 participants were included in the TUG analysis. Exercise-based interventions significantly reduced TUG completion time compared with control interventions (MD = −2.68 s, 95% CI: −3.72 to −1.65 s, *p* < 0.00001, I^2^ = 60%) (Figure 3).

**Figure 3:**
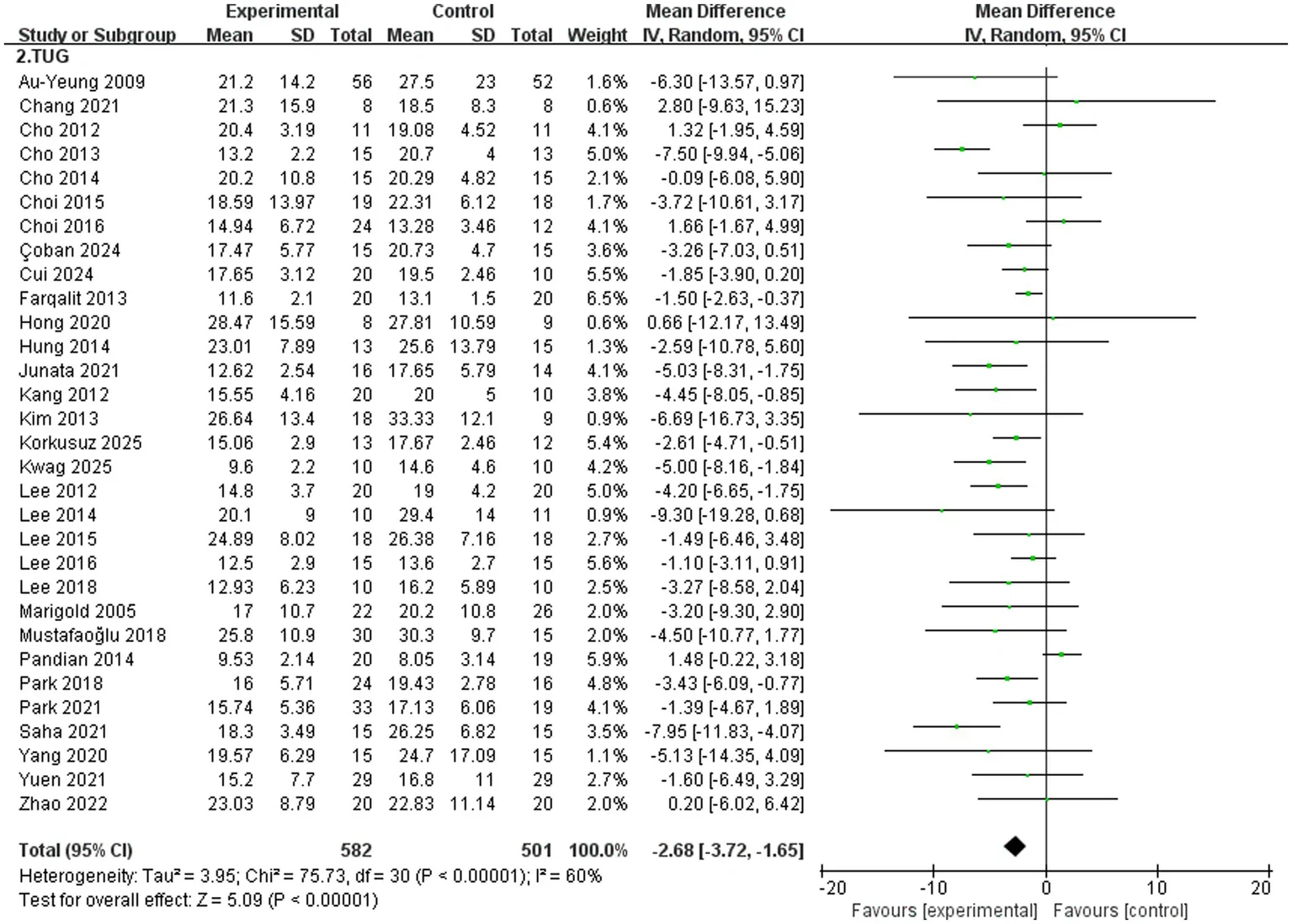
Forest plot showing the effects of exercise-based interventions on Timed Up and Go (TUG) performance in patients with chronic stroke. Data are presented as mean difference (MD) with 95% confidence intervals (CIs), calculated using a random-effects model.

#### FRT

Ten studies involving 295 participants were included in the FRT analysis. Exercise-based interventions significantly improved FRT performance compared with control interventions (SMD = 0.65, 95% CI: 0.25 to 1.05, *p* = 0.001, I^2^ = 62%) (Figure 4).

**Figure 4:**
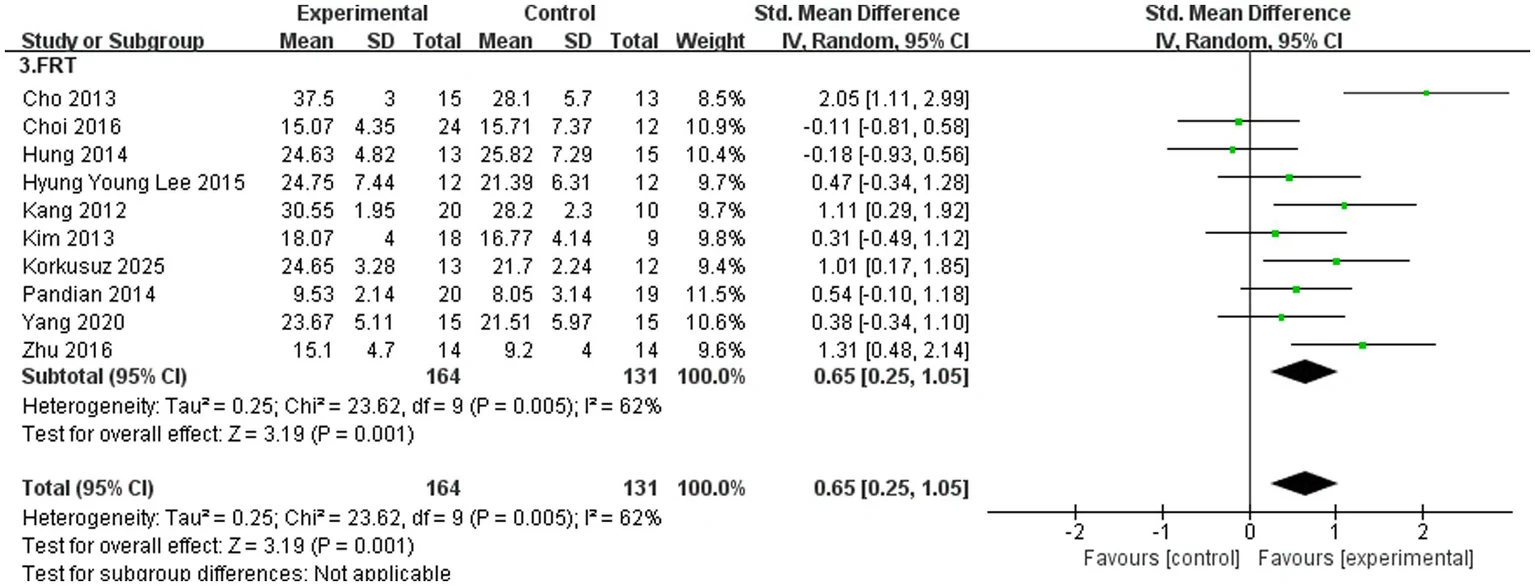
Forest plot showing the effects of exercise-based interventions on FRT performance in patients with chronic stroke. The Functional Reach Test (FRT) was used as the outcome measure. Data are presented as standardized mean difference (SMD) with 95% confidence intervals (CIs), calculated using a random-effects model.

### Sensitivity and subgroup analyses

Sensitivity analyses were conducted by sequentially excluding individual studies to assess the robustness of the pooled estimates. The overall effects for the BBS, TUG, and FRT outcomes remained consistent, indicating that no single study substantially influenced the direction or statistical significance of the pooled results.

Subgroup analyses were conducted according to the predefined intervention categories described in the Methods section when sufficient data were available. The corresponding subgroup results are presented in Supplementary Table S6, and subgroup forest plots for BBS and TUG are shown in Supplementary Figures S1, S2.

For the BBS outcome, balance or functional weight-shifting training (MD = 3.70, 95% CI: 1.77 to 5.63, I^2^ = 63%), gait training (MD = 2.92, 95% CI: 2.22 to 3.61, I^2^ = 0%), and other exercise modalities (MD = 4.63, 95% CI: 3.01 to 6.25, I^2^ = 0%) significantly improved BBS scores. In contrast, multisensory training (MD = 0.51, 95% CI: −0.57 to 1.59, I^2^ = 0%) and high-intensity aerobic training (MD = 0.32, 95% CI: −0.69 to 1.33, I^2^ = 0%) did not show significant effects. The between-subgroup difference was significant (Chi^2^ = 37.56, df = 4, *p* < 0.00001, I^2^ = 89.3%), suggesting that intervention type contributed to heterogeneity in BBS outcomes.

For the TUG outcome, gait training (MD = −3.16 s, 95% CI: −5.57 to −0.76, *p* = 0.01, I^2^ = 60%), multisensory training (MD = −2.54 s, 95% CI: −3.76 to −1.31, *p* < 0.0001, I^2^ = 0%), and other exercise modalities (MD = −3.59 s, 95% CI: −5.40 to −1.78, *p* = 0.0001, I^2^ = 55%) significantly reduced TUG completion time. In contrast, balance or functional weight-shifting training did not show a statistically significant effect (MD = −1.51 s; 95% CI: −3.91 to 0.88; *p* = 0.22; I^2^ = 63%). No statistically significant subgroup difference was observed for TUG (Chi^2^ = 2.08, df = 3, *p* = 0.56, I^2^ = 0%).

Although several TUG subgroups showed statistically significant pooled effects, the test for between-subgroup differences was not statistically significant. Therefore, the subgroup-specific estimates should be interpreted as descriptive trends rather than evidence that one intervention type was more effective than another.

Additional *post hoc* subgroup analyses were conducted according to comparator condition (Supplementary Figures S5–S7). For BBS, the pooled effects were MD = 3.59 (95% CI: 0.37 to 6.81; I^2^ = 0%) for minimal/no additional intervention, MD = 3.51 (95% CI: 1.97 to 5.05; I^2^ = 52%) for usual/routine rehabilitation, and MD = 2.09 (95% CI: 1.31 to 2.88; I^2^ = 43%) for active comparator interventions, with no statistically significant between-subgroup difference (Chi^2^ = 3.10, df = 2, *p* = 0.21). For TUG, the pooled effects were MD = −1.42 s (95% CI: −2.94 to 0.10; I^2^ = 65%) for usual/routine rehabilitation and MD = −3.76 s (95% CI: −5.07 to −2.44; I^2^ = 47%) for active comparator interventions; the between-subgroup difference was statistically significant (Chi^2^ = 5.17, df = 1, *p* = 0.02). For FRT, the pooled effects were SMD = 0.60 (95% CI: 0.06 to 1.14; I^2^ = 53%) for usual/routine rehabilitation and SMD = 0.69 (95% CI: 0.08 to 1.30; I^2^ = 71%) for active comparator interventions, with no statistically significant between-subgroup difference (Chi^2^ = 0.05, df = 1, *p* = 0.83).

Exploratory meta-regression showed no statistically significant association between intervention duration and effect estimates for the BBS (*β* = −0.02 per week, 95% CI − 0.24 to 0.20; *p* = 0.840), TUG (*β* = 0.10 per week, 95% CI − 0.30 to 0.50; *p* = 0.625), or FRT (*β* = −0.08 per week, 95% CI − 0.27 to 0.12; *p* = 0.404). Similarly, training frequency was not significantly associated with effect estimates for the BBS (*β* = 0.35 per additional session/week, 95% CI − 0.31 to 1.01; *p* = 0.292), TUG (*β* = 0.11 per additional session/week, 95% CI − 0.92 to 1.13; *p* = 0.833), or FRT (*β* = 0.18 per additional session/week, 95% CI − 0.27 to 0.63; *p* = 0.385). Intervention duration explained no measurable proportion of between-study heterogeneity (R^2^ = 0% for all outcomes), while training frequency explained little or no heterogeneity (R^2^ = 0.65% for BBS and 0% for TUG and FRT). Sensitivity analyses yielded materially unchanged BBS results (Supplementary Table S7).

Sensitivity analyses using three-level random-effects meta-analyses accounting for country-level clustering showed results consistent with the primary analyses. The pooled estimates remained similar for BBS (MD 2.52, 95% CI 1.45 to 3.58), TUG (MD − 2.69, 95% CI − 3.75 to −1.63), and FRT (SMD 0.65, 95% CI 0.25 to 1.06) (Supplementary Table S8).

### Publication bias

Funnel plots and Egger’s tests showed no significant evidence of publication bias for the TUG and FRT outcomes. However, Egger’s test suggested potential small-study effects for the BBS outcome (*p* = 0.009). Therefore, the BBS result should be interpreted with some caution, although sensitivity analyses supported the robustness of the pooled estimate. Funnel plots and Egger’s test results are presented in Supplementary Figures S3, S4, respectively.

### Certainty of evidence

The certainty of evidence was rated as moderate for the BBS outcome because Egger’s test detected potential small-study effects. The certainty of evidence for the TUG outcome was rated as high because no serious concerns were identified across the GRADE domains, and sensitivity analyses supported the robustness of the pooled estimate. For the FRT outcome, the certainty of the evidence was rated as moderate due to inconsistency related to moderate heterogeneity. The detailed GRADE evidence profile is presented in Supplementary Table S5.

## Discussion

This systematic review and meta-analysis included 69 randomized controlled trials involving 2,314 participants with chronic stroke from 16 countries. Overall, exercise-based interventions were associated with improvements in BBS, TUG, and FRT outcomes. However, interpretation varied by outcome: the BBS result was subject to potential small-study effects, TUG showed moderate heterogeneity, and FRT was based on fewer studies and a smaller sample with moderate heterogeneity. These findings should therefore be interpreted as specific to the outcome measures assessed and in light of outcome-specific certainty of evidence, as well as heterogeneity in interventions and comparator conditions.

The observed improvements in BBS, TUG, and FRT outcomes are clinically plausible because functional recovery in chronic stroke may involve improvements in residual motor capacity, postural control, and movement coordination rather than relying solely on spontaneous recovery processes. Even after the early recovery window, structured exercise may enhance functional performance by improving postural control, strengthening residual motor capacity, and promoting the coordination required for everyday movement (9). This interpretation is consistent with the outcome-specific findings of this review. The BBS and FRT mainly capture balance-related capacity, whereas the TUG reflects a more integrated mobility task involving sit-to-stand transfer, walking, turning, and sitting down (85). Therefore, interventions that closely match the functional demands assessed by a given outcome may be more likely to produce detectable improvements. In this sense, the effects of exercise-based interventions in chronic stroke depend not only on whether training is provided but also on how well the training content aligns with the functional domain being evaluated (10).

Although the included interventions shared the aim of improving balance and/or functional mobility through structured exercise, they varied in modality, dose, and delivery (8, 86). Comparator conditions ranged from no intervention or waiting-list control to usual care, conventional rehabilitation, sham intervention, and active rehabilitation and were therefore not clinically equivalent. Accordingly, the pooled estimates should be interpreted as average relative effects across heterogeneous interventions and comparator conditions, rather than as the effect of a single standardized exercise-based intervention program. The exploratory comparator-based subgroup analyses showed no statistically significant between-subgroup differences for BBS or FRT, whereas a statistically significant between-subgroup difference was observed for TUG. Given the *post hoc* nature of these analyses and potential differences in intervention content, dose, and other trial characteristics across subgroups, the TUG finding should be interpreted cautiously and does not establish that comparator type itself caused the observed difference.

Intervention-type subgroup analyses provided potential explanations for heterogeneity. For the BBS outcome, significant subgroup differences were observed across intervention types, suggesting that training content may influence improvements in BBS scores. For the TUG outcome, several intervention subgroups showed statistically significant pooled effects; however, the between-subgroup difference was not statistically significant, providing no evidence that one intervention type was more effective than another. For the FRT outcome, the limited number of included studies and residual heterogeneity restrict the interpretation of subgroup effects. Overall, subgroup analyses should be viewed as hypothesis-generating rather than as direct evidence that one exercise modality is superior to another (87). Differences in intervention characteristics, including training objectives and exercise components, may partly explain the variation in observed effects across outcomes. Intervention duration and training frequency varied across studies, but exploratory meta-regression showed no significant linear association between either characteristic and effect estimates for the BBS, TUG, or FRT. Given the study-level nature of these analyses and limited data for some outcomes, these findings should be interpreted cautiously.

The findings are broadly consistent with previous systematic reviews and rehabilitation guidelines indicating that structured exercise can improve balance and functional mobility after stroke (8, 88, 89). By restricting eligibility to chronic stroke, the present synthesis reduces the potential influence of spontaneous recovery and more specifically reflects rehabilitation-associated effects during this phase (90). The intervention subgroup analyses provide exploratory information but should not be interpreted as definitive comparative-effectiveness evidence (91).

From a clinical and public health perspective, persistent impairments in balance and functional mobility can reduce independence, increase fall risk and caregiver burden, and restrict social participation (5, 92). Most of the evidence synthesized in this review was derived from structured, therapist-supervised settings, with more limited evidence from home-based or partially supervised programs. Therefore, the findings are most directly applicable to structured exercise-based interventions, while their generalizability to less supervised community or home settings should be interpreted cautiously. Importantly, the present findings are specific to the BBS, TUG, and FRT and should not be interpreted as representing the full constructs of balance or functional mobility, as other validated measures may capture different dimensions and yield different estimates of intervention effects. Training should be aligned with the target outcome: postural control and weight-shifting exercises for balance and gait, turning, and sit-to-stand–walk–sit practice for functional mobility (10, 92).

This review has several strengths, including its focus on adults with chronic stroke, the inclusion of 69 randomized controlled trials, and the synthesis of complementary balance and functional mobility outcomes using the BBS, TUG, and FRT (85). Subgroup and sensitivity analyses were conducted, and the PEDro scale and GRADE approach were used to support transparent assessment and interpretation of the evidence (93, 94).

Several limitations should also be considered. Although this review focused on adults with chronic stroke, some included studies reported stroke duration using group-level means or ranges, and the chronicity of every individual participant could not always be fully verified. Residual clinical heterogeneity related to time since stroke may therefore remain. In addition, variation in intervention characteristics and non-equivalent comparator conditions may have contributed to heterogeneity and influenced the pooled effect estimates. Although three-level sensitivity analyses accounting for country-level clustering yielded estimates consistent with the primary analyses, residual heterogeneity may still reflect differences across study settings, intervention characteristics, and participant populations. Some subgroups included only a small number of studies, limiting the stability of the subgroup findings. Furthermore, only English-language articles were included, and studies with PEDro scores below 4 were excluded; therefore, potential language bias and reduced comprehensiveness related to the quality-based exclusion criterion cannot be completely ruled out. Finally, many studies inadequately reported training intensity, progression principles, adherence, adverse events, and long-term follow-up, making it difficult to determine the optimal exercise type, dose, implementation strategy, and durability of effects (10, 95). Recent Cochrane evidence on cardiorespiratory training after stroke also suggests potential benefits for balance and walking-related outcomes, further supporting the role of exercise-based interventions in post-stroke rehabilitation (96).

In summary, this systematic review and meta-analysis indicate that exercise-based interventions may improve BBS, TUG, and FRT outcomes in adults with chronic stroke. Intervention-type subgroup analyses suggested possible differences for BBS, whereas no statistically significant between-subgroup difference across intervention types was observed for TUG. Accordingly, the current evidence does not establish the superiority of any specific exercise modality. Future trials should use rigorous designs, standardized intervention reporting, and adequate follow-up to clarify optimal training components, dose, delivery, and durability.

## Data Availability

All data analyzed in this systematic review and meta-analysis were extracted from previously published studies, which are publicly available through the original publications and their Supplementary materials. The extracted dataset and statistical code used for the current meta-analysis are available from the corresponding author upon reasonable request.
