# Supplementary material for: Effects of exercise-based interventions on balance and functional mobility in adults with chronic stroke: a systematic review and meta-analysis
Source: Front Public Health. 2026 Oct 13;14:1930987. doi: 10.3389/fpubh.2026.1930987 (PMC13710241; doi:10.3389/fpubh.2026.1930987)
Supplement: Supplementary file 1 [file Supplementary_file_1.docx]

***Supplementary Material***

**Supplementary Table S1.** **PRISMA 2020 Checklist**

| **Section and Topic** | **Item #** | **Checklist item** | **Location where item is reported** |
| --- | --- | --- | --- |
| **TITLE** | | |  |
| Title | 1 | Identify the report as a systematic review. | Title |
| **ABSTRACT** | | |  |
| Abstract | 2 | See the PRISMA 2020 for Abstracts checklist. | Abstract |
| **INTRODUCTION** | | |  |
| Rationale | 3 | Describe the rationale for the review in the context of existing knowledge. | Introduction |
| Objectives | 4 | Provide an explicit statement of the objective(s) or question(s) the review addresses. | Introduction |
| **METHODS** | | |  |
| Eligibility criteria | 5 | Specify the inclusion and exclusion criteria for the review and how studies were grouped for the syntheses. | Methods |
| Information sources | 6 | Specify all databases, registers, websites, organisations, reference lists and other sources searched or consulted to identify studies. Specify the date when each source was last searched or consulted. | Methods |
| Search strategy | 7 | Present the full search strategies for all databases, registers and websites, including any filters and limits used. | Methods; Supplementary Table S2 |
| Selection process | 8 | Specify the methods used to decide whether a study met the inclusion criteria of the review, including how many reviewers screened each record and each report retrieved, whether they worked independently, and if applicable, details of automation tools used in the process. | Methods; Figure 1 |
| Data collection process | 9 | Specify the methods used to collect data from reports, including how many reviewers collected data from each report, whether they worked independently, any processes for obtaining or confirming data from study investigators, and if applicable, details of automation tools used in the process. | Methods |
| Data items | 10a | List and define all outcomes for which data were sought. Specify whether all results that were compatible with each outcome domain in each study were sought (e.g. for all measures, time points, analyses), and if not, the methods used to decide which results to collect. | Methods |
|  | 10b | List and define all other variables for which data were sought (e.g. participant and intervention characteristics, funding sources). Describe any assumptions made about any missing or unclear information. | Methods; Table 1 |
| Study risk of bias assessment | 11 | Specify the methods used to assess risk of bias in the included studies, including details of the tool(s) used, how many reviewers assessed each study and whether they worked independently, and if applicable, details of automation tools used in the process. | Methods; Supplementary Table S4 |
| Effect measures | 12 | Specify for each outcome the effect measure(s) (e.g. risk ratio, mean difference) used in the synthesis or presentation of results. | Methods |
| Synthesis methods | 13a | Describe the processes used to decide which studies were eligible for each synthesis (e.g. tabulating the study intervention characteristics and comparing against the planned groups for each synthesis (item #5)). | Methods |
|  | 13b | Describe any methods required to prepare the data for presentation or synthesis, such as handling of missing summary statistics, or data conversions. | Methods |
|  | 13c | Describe any methods used to tabulate or visually display results of individual studies and syntheses. | Methods; Figures 2–4 |
|  | 13d | Describe any methods used to synthesize results and provide a rationale for the choice(s). If meta-analysis was performed, describe the model(s), method(s) to identify the presence and extent of statistical heterogeneity, and software package(s) used. | Methods |
|  | 13e | Describe any methods used to explore possible causes of heterogeneity among study results (e.g. subgroup analysis, meta-regression). | Methods; Supplementary Tables S6–S7 |
|  | 13f | Describe any sensitivity analyses conducted to assess robustness of the synthesized results. | Methods; Supplementary Table S7 |
| Reporting bias assessment | 14 | Describe any methods used to assess risk of bias due to missing results in a synthesis (arising from reporting biases). | Methods; Supplementary Figures S3–S4 |
| Certainty assessment | 15 | Describe any methods used to assess certainty (or confidence) in the body of evidence for an outcome. | Methods; Supplementary Table S5 |
| **RESULTS** | | |  |
| Study selection | 16a | Describe the results of the search and selection process, from the number of records identified in the search to the number of studies included in the review, ideally using a flow diagram. | Results; Figure 1 |
|  | 16b | Cite studies that might appear to meet the inclusion criteria, but which were excluded, and explain why they were excluded. | Results; Supplementary Table S3 |
| Study characteristics | 17 | Cite each included study and present its characteristics. | Results;  Table 1 |
| Risk of bias in studies | 18 | Present assessments of risk of bias for each included study. | Results; Supplementary Table S4 |
| Results of individual studies | 19 | For all outcomes, present, for each study: (a) summary statistics for each group (where appropriate) and (b) an effect estimate and its precision (e.g. confidence/credible interval), ideally using structured tables or plots. | Results; Figures 2–4 |
| Results of syntheses | 20a | For each synthesis, briefly summarise the characteristics and risk of bias among contributing studies. | Results; Supplementary Table S4 |
|  | 20b | Present results of all statistical syntheses conducted. If meta-analysis was done, present for each the summary estimate and its precision (e.g. confidence/credible interval) and measures of statistical heterogeneity. If comparing groups, describe the direction of the effect. | Results; Figures 2–4 |
|  | 20c | Present results of all investigations of possible causes of heterogeneity among study results. | Results; Supplementary Tables S6–S7; Supplementary Figures S1–S2, S5–S7 |
|  | 20d | Present results of all sensitivity analyses conducted to assess the robustness of the synthesized results. | Results; Supplementary Table S7 |
| Reporting biases | 21 | Present assessments of risk of bias due to missing results (arising from reporting biases) for each synthesis assessed. | Results; Supplementary Figures S3–S4 |
| Certainty of evidence | 22 | Present assessments of certainty (or confidence) in the body of evidence for each outcome assessed. | Results; Supplementary Table S5 |
| **DISCUSSION** | | |  |
| Discussion | 23a | Provide a general interpretation of the results in the context of other evidence. | Discussion |
|  | 23b | Discuss any limitations of the evidence included in the review. | Discussion |
|  | 23c | Discuss any limitations of the review processes used. | Discussion |
|  | 23d | Discuss implications of the results for practice, policy, and future research. | Discussion |
| **OTHER INFORMATION** | | |  |
| Registration and protocol | 24a | Provide registration information for the review, including register name and registration number, or state that the review was not registered. | Methods |
|  | 24b | Indicate where the review protocol can be accessed, or state that a protocol was not prepared. | Methods |
|  | 24c | Describe and explain any amendments to information provided at registration or in the protocol. | Not applicable |
| Support | 25 | Describe sources of financial or non-financial support for the review, and the role of the funders or sponsors in the review. | Funding |
| Competing interests | 26 | Declare any competing interests of review authors. | Conflict of interest |
| Availability of data, code and other materials | 27 | Report which of the following are publicly available and where they can be found: template data collection forms; data extracted from included studies; data used for all analyses; analytic code; any other materials used in the review. | Data availability statement |

*From:* Page MJ, McKenzie JE, Bossuyt PM, Boutron I, Hoffmann TC, Mulrow CD, et al. The PRISMA 2020 statement: an updated guideline for reporting systematic reviews. BMJ 2021;372:n71. doi: 10.1136/bmj.n71

For more information, visit: <http://www.prisma-statement.org/>

**Supplementary Table S2: Detailed description of the search strategy (29 June 2025)**

| **PubMed** | |
| --- | --- |
| #1 | "Stroke"[MeSH Terms] OR "Brain Ischemia"[MeSH Terms] OR "Cerebral Infarction"[MeSH Terms] OR stroke[Title/Abstract] OR strokes[Title/Abstract] OR poststroke[Title/Abstract] OR post-stroke[Title/Abstract] OR "cerebrovascular accident*"[Title/Abstract] OR "cerebral vascular accident*"[Title/Abstract] OR "cerebrovascular disorder*"[Title/Abstract] OR "brain ischemia"[Title/Abstract] OR "brain ischaemia"[Title/Abstract] OR "cerebral infarction"[Title/Abstract] OR "cerebral infarct*"[Title/Abstract] |
| #2 | "Exercise Therapy"[MeSH Terms] OR "Exercise"[MeSH Terms] OR "Physical Therapy Modalities"[MeSH Terms] OR "Rehabilitation"[MeSH Terms] OR "Motor Activity"[MeSH Terms] OR "Postural Balance"[MeSH Terms] OR "Gait"[MeSH Terms] OR exercise[Title/Abstract] OR exercises[Title/Abstract] OR "exercise therapy"[Title/Abstract] OR "physical therapy"[Title/Abstract] OR physiotherapy[Title/Abstract] OR rehabilitation[Title/Abstract] OR training[Title/Abstract] OR "balance training"[Title/Abstract] OR balance[Title/Abstract] OR gait[Title/Abstract] OR walking[Title/Abstract] OR mobility[Title/Abstract] OR "motor training"[Title/Abstract] OR "task-oriented training"[Title/Abstract] OR "task-specific training"[Title/Abstract] OR "aerobic training"[Title/Abstract] OR "resistance training"[Title/Abstract] OR strengthening[Title/Abstract] |
| #3 | "Randomized Controlled Trial"[Publication Type] OR "Controlled Clinical Trial"[Publication Type] OR "Randomized Controlled Trials as Topic"[MeSH Terms] OR randomized[Title/Abstract] OR randomised[Title/Abstract] OR randomly[Title/Abstract] OR "randomized controlled trial"[Title/Abstract] OR "randomised controlled trial"[Title/Abstract] OR "randomized clinical trial"[Title/Abstract] OR "randomised clinical trial"[Title/Abstract] OR RCT[Title/Abstract] OR trial[Title/Abstract] |
| #4 | #1 AND #2 AND #3 |
| #5 | "Review"[Publication Type] OR "Letter"[Publication Type] OR "Editorial"[Publication Type] OR "Comment"[Publication Type] |
| #6 | #4 NOT #5 |
| **Web of Science** | |
| #1 | TS=(stroke* OR poststroke OR "post-stroke" OR "cerebrovascular accident*" OR "cerebral vascular accident*" OR "cerebrovascular disorder*" OR "brain ischemia" OR "brain ischaemia" OR "cerebral ischemia" OR "cerebral ischaemia" OR "cerebral infarction" OR "cerebral infarct*") |
| #2 | TS=("exercise therapy" OR exercise* OR "physical therap*" OR physiotherap* OR rehabilitation OR "motor activity" OR "physical activity" OR training OR "balance training" OR balance OR "postural balance" OR gait OR walking OR mobility OR "gait training" OR "motor training" OR "task-oriented training" OR "task-specific training" OR "aerobic training" OR "resistance training" OR strengthening) |
| #3 | TS=("randomized controlled trial" OR "randomised controlled trial" OR "randomized clinical trial" OR "randomised clinical trial" OR RCT OR randomized OR randomised OR randomly OR trial*) |
| #4 | #1 AND #2 AND #3 |
| #5 | DT=(Review OR Editorial Material OR Letter OR Meeting Abstract OR Book Chapter) |
| #6 | #4 NOT #5 |
| **EMBASE** | |
| #1 | 'cerebrovascular accident'/exp OR 'stroke'/exp OR 'brain ischemia'/exp OR 'cerebral infarction'/exp OR stroke*:ti,ab OR poststroke:ti,ab OR 'post-stroke':ti,ab OR 'cerebrovascular accident*':ti,ab OR 'cerebral vascular accident*':ti,ab OR 'cerebrovascular disorder*':ti,ab OR 'cerebrovascular disease*':ti,ab OR 'brain ischemia':ti,ab OR 'brain ischaemia':ti,ab OR 'cerebral ischemia':ti,ab OR 'cerebral ischaemia':ti,ab OR 'cerebral infarction':ti,ab OR 'cerebral infarct*':ti,ab |
| #2 | 'exercise'/exp OR 'exercise therapy'/exp OR 'physical therapy'/exp OR 'rehabilitation'/exp OR 'motor activity'/exp OR 'balance'/exp OR 'gait'/exp OR exercise*:ti,ab OR 'exercise therapy':ti,ab OR 'physical therap*':ti,ab OR physiotherap*:ti,ab OR rehabilitation:ti,ab OR training:ti,ab OR 'balance training':ti,ab OR balance:ti,ab OR 'postural balance':ti,ab OR gait:ti,ab OR walking:ti,ab OR mobility:ti,ab OR 'gait training':ti,ab OR 'motor training':ti,ab OR 'task-oriented training':ti,ab OR 'task-specific training':ti,ab OR 'aerobic training':ti,ab OR 'resistance training':ti,ab OR strengthening:ti,ab OR 'physical activity':ti,ab |
| #3 | 'randomized controlled trial'/exp OR 'controlled clinical trial'/exp OR random*:ti,ab OR randomised:ti,ab OR randomized:ti,ab OR randomly:ti,ab OR 'randomized controlled trial':ti,ab OR 'randomised controlled trial':ti,ab OR 'randomized clinical trial':ti,ab OR 'randomised clinical trial':ti,ab OR rct:ti,ab OR trial*:ti,ab |
| #4 | #1 AND #2 AND #3 |
| #5 | 'review'/it OR 'letter'/it OR 'editorial'/it OR 'conference abstract'/it OR 'conference paper'/it |
| #6 | #4 NOT #5 |
| **Scopus** | |
| #1 | TITLE-ABS-KEY(stroke* OR poststroke OR "post-stroke" OR "cerebrovascular accident*" OR "cerebral vascular accident*" OR "cerebrovascular disorder*" OR "cerebrovascular disease*" OR "brain ischemia" OR "brain ischaemia" OR "cerebral ischemia" OR "cerebral ischaemia" OR "cerebral infarction" OR "cerebral infarct*") |
| #2 | TITLE-ABS-KEY("exercise therapy" OR exercise* OR "physical therap*" OR physiotherap* OR rehabilitation OR "motor activity" OR "physical activity" OR training OR "balance training" OR balance OR "postural balance" OR gait OR walking OR mobility OR "gait training" OR "motor training" OR "task-oriented training" OR "task-specific training" OR "aerobic training" OR "resistance training" OR strengthening) |
| #3 | TITLE-ABS-KEY("randomized controlled trial" OR "randomised controlled trial" OR "randomized clinical trial" OR "randomised clinical trial" OR "randomized trial" OR "randomised trial" OR RCT OR random* OR randomly OR trial*) |
| #4 | #1 AND #2 AND #3 |
| #5 | #4 AND NOT (DOCTYPE(re) OR DOCTYPE(ed) OR DOCTYPE(le) OR DOCTYPE(cp) OR DOCTYPE(ch)) |

**Supplementary Table S3. Studies excluded (n=52) with reasons**

| **Studies excluded** | **Reasons** |
| --- | --- |
| Zandvliet, 2019 [1] | The study type is wrong(protocol) |
| Gabriela, 2019 [2] | The study type is wrong(protocol) |
| Ten, 2015 [3] | The study type is wrong(protocol) |
| Mandiroglu, 2022 [4] | The study type is wrong(Cross-sectional study) |
| Khan, 2022 [5] | The study type is wrong(Cross-sectional study) |
| Gath, 2021 [6] | The study type is wrong(Cross-sectional study) |
| Van, 2024 [7] | The study type is wrong(Meeting abstract) |
| Maciaszek, 2018 [8] | No data available |
| Karthikbabu, 2018 [9] | No data available |
| Emília, 2017 [10] | No data available |
| Lee, 2017 [11] | No data available |
| Lisiński, 2012 [12] | No data available |
| Lou, 2010 [13] | No data available |
| Mansfield, 2010 [14] | No data available |
| Eser, 2008 [15] | No data available |
| Sana, 2023 [16] | Stroke less than six months old |
| Mihara, 2021[17] | Stroke less than six months old |
| Noh, 2019 [18] | Stroke less than six months old |
| Kim, 2019 [19] | Stroke less than six months old |
| Kal, 2019 [20] | Stroke less than six months old |
| Lee, 2018 [21] | Stroke less than six months old |
| Kim, 2018 [22] | Stroke less than six months old |
| Graham,2018[23] | Stroke less than six months old |
| Golla, 2018 [24] | Stroke less than six months old |
| Elsner, 2018 [25] | Stroke less than six months old |
| Vahlberg,2017 [26] | Stroke less than six months old |
| Goljar, 2010 [27] | Stroke less than six months old |
| Onigbinde, 2009 [28] | Stroke less than six months old |
| Katz-Leurer, 2006 [29] | Stroke less than six months old |
| Lau, 2011 [30] | Stroke less than six months old |
| Bei, 2023 [31] | Stroke less than six months old |
| Lee, 2020 [32] | Stroke less than six months old |
| Cabanas-Valdés, 2016 [33] | Stroke less than six months old |
| Wu, 2020 [34] | Stroke less than six months old |
| Lee, 2023 [35] | Stroke less than six months old |
| Díaz-López, 2022 [36] | Stroke less than six months old |
| Haleem, 2025 [37] | Stroke less than six months old |
| Sheehy, 2020 [38] | Stroke less than six months old |
| Mauro, 2024 [39] | Stroke less than six months old |
| Qurat, 2018 [40] | Stroke less than six months old |
| Cannell, 2018 [41] | Stroke less than six months old |
| Mackay, 2013 [42] | Stroke less than six months old |
| Calisgan, 2024 [43] | Stroke less than six months old |
| Almasoudi, 2024 [44] | Stroke less than six months old |
| Cho, 2020 [45] | Low-quality literature |
| Fritz, 2013 [46] | Low-quality literature |
| In,2016[47] | Low-quality literature |
| Hong,2024[48] | Low-quality literature |
| Madhuranga,2019[49] | Low-quality literature |
| Merkert,2011[50] | Low-quality literature |
| Jacobson,2011[51] | No data available |
| Yaşa, 2022[52] | No data available |

(1) Zandvliet SB, Meskers CG, Nijland RH, Daffertshofer A, Kwakkel G, van Wegen EE. The effect of cerebellar transcranial direct current stimulation to improve standing balance performance early post-stroke, study protocol of a randomized controlled trial. Int J Stroke. 2019 Aug;14(6):650-657.

(2) Gabriela de Paula GV, da Silva TR, de Souza JT, Luvizutto GJ, Bazan SGZ, Modolo GP, Winckler FC, de Oliveira Antunes LC, Martin LC, da Costa RDM, Bazan R. Effect of ankle-foot orthosis on functional mobility and dynamic balance of patients after stroke: Study protocol for a randomized controlled clinical trial. Medicine (Baltimore). 2019 Sep;98(39):e17317.

(3) Ten Brink AF, Visser-Meily JM, Nijboer TC. Study protocol of 'Prism Adaptation in Rehabilitation': a randomized controlled trial in stroke patients with neglect. BMC Neurol. 2015 Feb 4;15:5.

(4) Mandiroglu S, Firat M. Foot and ankle deformities in stroke: relationship with ambulation, balance, and daily living activities: a cross-sectional study. Somatosens Mot Res. 2022 Jun-Dec;39(2-4):106-110.

(5) Khan F, Abusharha S, Alfuraidy A, Nimatallah K, Almalki R, Basaffar R, Mirdad M, Chevidikunnan MF, Basuodan R. Prediction of Factors Affecting Mobility in Patients with Stroke and Finding the Mediation Effect of Balance on Mobility: A Cross-Sectional Study. Int J Environ Res Public Health. 2022 Dec 10;19(24):16612.

(6) Gath CF, Gianella MG, Bonamico L, Olmos L, Russo MJ. Prediction of Balance After Inpatient Rehabilitation in Stroke Subjects with Severe Balance Alterations at the Admission. J Stroke Cerebrovasc Dis. 2021 Apr;30(4):105627.

(7) Van Criekinge T, Heremans C, Burridge J, Deutsch JE, Hammerbeck U, Hollands K, Karthikbabu S, Mehrholz J, Moore JL, Salbach NM, Schröder J, Veerbeek JM, Weerdesteyn V, Borschmann K, Churilov L, Verheyden G, Kwakkel G. Standardized measurement of balance and mobility post-stroke: Consensus-based core recommendations from the third Stroke Recovery and Rehabilitation Roundtable. Int J Stroke. 2024 Feb;19(2):158-168.

(8) Maciaszek J. Effects of Posturographic Platform Biofeedback Training on the Static and Dynamic Balance of Older Stroke Patients. J Stroke Cerebrovasc Dis. 2018 Jul;27(7):1969-1974.

(9) Karthikbabu S, Chakrapani M, Ganesan S, Ellajosyula R, Solomon JM. Efficacy of Trunk Regimes on Balance, Mobility, Physical Function, and Community Reintegration in Chronic Stroke: A Parallel-Group Randomized Trial. J Stroke Cerebrovasc Dis. 2018 Apr;27(4):1003-1011.

(10) E Silva EMGS, Ribeiro TS, da Silva TCC, Costa MFP, Cavalcanti FADC, Lindquist ARR. Effects of constraint-induced movement therapy for lower limbs on measurements of functional mobility and postural balance in subjects with stroke: a randomized controlled trial. Top Stroke Rehabil. 2017 Dec;24(8):555-561.

(11) Lee HC, Huang CL, Ho SH, Sung WH. The Effect of a Virtual Reality Game Intervention on Balance for Patients with Stroke: A Randomized Controlled Trial. Games Health J. 2017 Oct;6(5):303-311.

(12) Lisiński P, Huber J, Gajewska E, Szłapiński P. The body balance training effect on improvement of motor functions in paretic extremities in patients after stroke. A randomized, single blinded trial. Clin Neurol Neurosurg. 2012 Jan;114(1):31-6.

(13) Lou BD, Zhang W, Liu Z, Lan H, Li JR, Wang JJ. [Clinical evaluation on balanced muscular tension needling method for improving disabled function of stroke patients with spastic paralysis]. Zhongguo Zhen Jiu. 2010 Feb;30(2):89-92.

(14) Mansfield A, Peters AL, Liu BA, Maki BE. Effect of a perturbation-based balance training program on compensatory stepping and grasping reactions in older adults: a randomized controlled trial. Phys Ther. 2010 Apr;90(4):476-91.

(15) Eser F, Yavuzer G, Karakus D, Karaoglan B. The effect of balance training on motor recovery and ambulation after stroke: a randomized controlled trial. Eur J Phys Rehabil Med. 2008 Mar;44(1):19-25.

(16) Sana V, Ghous M, Kashif M, Albalwi A, Muneer R, Zia M. Effects of vestibular rehabilitation therapy versus virtual reality on balance, dizziness, and gait in patients with subacute stroke: A randomized controlled trial. Medicine (Baltimore). 2023 Jun 16;102(24):e33203.

(17) Mihara M, Fujimoto H, Hattori N, Otomune H, Kajiyama Y, Konaka K, Watanabe Y, Hiramatsu Y, Sunada Y, Miyai I, Mochizuki H. Effect of Neurofeedback Facilitation on Poststroke Gait and Balance Recovery: A Randomized Controlled Trial. Neurology. 2021 May 25;96(21):e2587-e2598.

(18) Noh HJ, Lee SH, Bang DH. Three-Dimensional Balance Training Using Visual Feedback on Balance and Walking Ability in Subacute Stroke Patients: A Single-Blinded Randomized Controlled Pilot Trial. J Stroke Cerebrovasc Dis. 2019 Apr;28(4):994-1000.

(19) Kim HY, Shin JH, Yang SP, Shin MA, Lee SH. Robot-assisted gait training for balance and lower extremity function in patients with infratentorial stroke: a single-blinded randomized controlled trial. J Neuroeng Rehabil. 2019 Jul 29;16(1):99. doi: 10.1186/s12984-019-0553-5. PMID: 31358017; PMCID: PMC6664752.

(20) Kal E, Houdijk H, van der Kamp J, Verhoef M, Prosée R, Groet E, Winters M, van Bennekom C, Scherder E. Are the effects of internal focus instructions different from external focus instructions given during balance training in stroke patients? A double-blind randomized controlled trial. Clin Rehabil. 2019 Feb;33(2):207-221.

(21) Lee MM, Lee KJ, Song CH. Game-Based Virtual Reality Canoe Paddling Training to Improve Postural Balance and Upper Extremity Function: A Preliminary Randomized Controlled Study of 30 Patients with Subacute Stroke. Med Sci Monit. 2018 Apr 27;24:2590-2598.

(22) Kim SL, Lee BH. The Effects of Posterior Talar Glide and Dorsiflexion of the Ankle Plus Mobilization with Movement on Balance and Gait Function in Patient with Chronic Stroke: A Randomized Controlled Trial. J Neurosci Rural Pract. 2018 Jan-Mar;9(1):61-67.

(23) Graham SA, Roth EJ, Brown DA. Walking and balance outcomes for stroke survivors: a randomized clinical trial comparing body-weight-supported treadmill training with versus without challenging mobility skills. J Neuroeng Rehabil. 2018 Nov 1;15(1):92.

(24) Golla A, Müller T, Wohlfarth K, Jahn P, Mattukat K, Mau W. Home-based balance training using Wii Fit™: a pilot randomised controlled trial with mobile older stroke survivors. Pilot Feasibility Stud. 2018 Aug 25;4:143.

(25) Elsner B, Schweder S, Mehrholz J. Immediate effects of rest periods on balance control in patients after stroke. A randomized controlled pilot trial. BMC Res Notes. 2018 May 24;11(1):338.

(26) Vahlberg B, Cederholm T, Lindmark B, Zetterberg L, Hellström K. Short-term and long-term effects of a progressive resistance and balance exercise program in individuals with chronic stroke: a randomized controlled trial. Disabil Rehabil. 2017 Aug;39(16):1615-1622.

(27) Goljar N, Burger H, Rudolf M, Stanonik I. Improving balance in subacute stroke patients: a randomized controlled study. Int J Rehabil Res. 2010 Sep;33(3):205-10.

(28) Onigbinde AT, Awotidebe T, Awosika H. Effect of 6 weeks wobble board exercises on static and dynamic balance of stroke survivors. Technol Health Care. 2009;17(5-6):387-92.

(29) Katz-Leurer M, Sender I, Keren O, Dvir Z. The influence of early cycling training on balance in stroke patients at the subacute stage. Results of a preliminary trial. Clin Rehabil. 2006 May;20(5):398-405.

(30) Lau KW, Mak MK. Speed-dependent treadmill training is effective to improve gait and balance performance in patients with sub-acute stroke. J Rehabil Med. 2011 Jul;43(8):709-13.

(31) Bei N, Long D, Bei Z, Chen Y, Chen Z, Xing Z. Effect of Water Exercise Therapy on Lower Limb Function Rehabilitation in Hemiplegic Patients with the First Stroke. Altern Ther Health Med. 2023 Oct;29(7):429-433.

(32) Lee J, Jeon J, Lee D, Hong J, Yu J, Kim J. Effect of trunk stabilization exercise on abdominal muscle thickness, balance and gait abilities of patients with hemiplegic stroke: A randomized controlled trial. NeuroRehabilitation. 2020;47(4):435-442.

(33) Cabanas-Valdés R, Bagur-Calafat C, Girabent-Farrés M, Caballero-Gómez FM, Hernández-Valiño M, Urrútia Cuchí G. The effect of additional core stability exercises on improving dynamic sitting balance and trunk control for subacute stroke patients: a randomized controlled trial. Clin Rehabil. 2016 Oct;30(10):1024-1033.

(34) Wu WX, Zhou CY, Wang ZW, Chen GQ, Chen XL, Jin HM, He DR. Effect of Early and Intensive Rehabilitation after Ischemic Stroke on Functional Recovery of the Lower Limbs: A Pilot, Randomized Trial. J Stroke Cerebrovasc Dis. 2020 May;29(5):104649.

(35) Lee J, Kim DY, Lee SH, Kim JH, Kim DY, Lim KB, Yoo J. End-effector lower limb robot-assisted gait training effects in subacute stroke patients: A randomized controlled pilot trial. Medicine (Baltimore). 2023 Oct 20;102(42):e35568.

(36) Díaz-López N, Monge-Pereira E, Jodra-Centeno E, Molina-Rueda F, Miangolarra-Page JC. Use of recognition of laterality through implicit motor imagery for the improvement of postural control and balance in subacute stroke patients: a randomized controlled study. Rev Neurol. 2022 Jun 16;74(12):375-382. English, Spanish.

(37) Haleem MH, Baig MO, Abualait T, Yoo WK, Obaid S, Bashir S. Effects of transcranial direct current stimulation combined with motor relearning program on strength and balance in stroke patients. PeerJ. 2025 Feb 19;13:e18925.

(38) Sheehy L, Taillon-Hobson A, Sveistrup H, Bilodeau M, Yang C, Finestone H. Sitting Balance Exercise Performed Using Virtual Reality Training on a Stroke Rehabilitation Inpatient Service: A Randomized Controlled Study. PM R. 2020 Aug;12(8):754-765.

(39) Mauro MC, Fasano A, Germanotta M, Cortellini L, Insalaco S, Pavan A, Comanducci A, Guglielmelli E, Aprile IG. Restoring of Interhemispheric Symmetry in Patients With Stroke Following Bilateral or Unilateral Robot-Assisted Upper-Limb Rehabilitation: A Pilot Randomized Controlled Trial. IEEE Trans Neural Syst Rehabil Eng. 2024;32:3590-3602.

(40) Qurat-ul-Ain; Malik AN, Amjad I. Effect of circuit gait training vs traditional gait training on mobility performance in stroke. J Pak Med Assoc. 2018 Mar;68(3):455-458.

(41) Cannell J, Jovic E, Rathjen A, Lane K, Tyson AM, Callisaya ML, Smith ST, Ahuja KD, Bird ML. The efficacy of interactive, motion capture-based rehabilitation on functional outcomes in an inpatient stroke population: a randomized controlled trial. Clin Rehabil. 2018 Feb;32(2):191-200.

(42) Mackay-Lyons M, McDonald A, Matheson J, Eskes G, Klus MA. Dual effects of body-weight supported treadmill training on cardiovascular fitness and walking ability early after stroke: a randomized controlled trial. Neurorehabil Neural Repair. 2013 Sep;27(7):644-53.

(43) Calisgan E, Talu B. The effect of vestibular and somatosensory rehabilitation in addition to early rehabilitation on balance after stroke: a randomized controlled trial. Top Stroke Rehabil. 2024 Oct;31(7):703-712.

(44) Almasoudi AO, Seyam MK, Sanchez F. The effect of trunk exercises with hip strategy training to maximize independence level and balance for patient with stroke: Randomized controlled study. Physiother Res Int. 2024 Oct;29(4):e2142.

(45) Cho H, Kim K. Effects of Action Observation Training with Auditory Stimulation on Static and Dynamic Balance in Chronic Stroke Patients. J Stroke Cerebrovasc Dis. 2020 May;29(5):104775.

(46) Fritz SL, Peters DM, Merlo AM, Donley J. Active video-gaming effects on balance and mobility in individuals with chronic stroke: a randomized controlled trial. Top Stroke Rehabil. 2013 May-Jun;20(3):218-25.

(47) In T, Lee K, Song C. Virtual Reality Reflection Therapy Improves Balance and Gait in Patients with Chronic Stroke: Randomized Controlled Trials. Med Sci Monit. 2016 Oct 28;22:4046-4053.

(48) Hong X, Li S, Zhong Z, Lin Y, Zhang K. Effects of acupuncture combined with trunk strengthening training on balance and gait abilities in stroke hemiplegic patients. Medicine (Baltimore). 2024 Jul 19;103(29):e37784.

(49) Madhuranga PVH, Mathangasinghe Y, Anthony DJ. Improving balance with wobble board exercises in stroke patients: single-blind, randomized clinical trial. Top Stroke Rehabil. 2019 Dec;26(8):595-601.

(50) Merkert J, Butz S, Nieczaj R, Steinhagen-Thiessen E, Eckardt R. Combined whole body vibration and balance training using Vibrosphere®: improvement of trunk stability, muscle tone, and postural control in stroke patients during early geriatric rehabilitation. Z Gerontol Geriatr. 2011 Aug;44(4):256-61.

(51) Jacobson BH, Thompson B, Wallace T, Brown L, Rial C. Independent static balance training contributes to increased stability and functional capacity in community-dwelling elderly people: a randomized controlled trial. Clin Rehabil. 2011 Jun;25(6):549-56.

(52) Yaşa ME, Özkan T, Ünlüer NÖ, Çelenay ŞT, Anlar Ö. Core stability-based balance training and kinesio taping for balance, trunk control, fear of falling and walking capacity in patients with multiple sclerosis: A randomized single-blinded study. Mult Scler Relat Disord. 2022 Dec;68:104178.

**Supplementary Table S4. Quality assessment of included studies**

| **Author(Publication Year)** | **Physiotherapy Evidence Database (PEDro) Scale** | | | | | | | | | | | |
| --- | --- | --- | --- | --- | --- | --- | --- | --- | --- | --- | --- | --- |
|  | **a** | **b** | **c** | **d** | **e** | **f** | **g** | **h** | **i** | **j** | **k** | **Total** |
| Saha 2021 | 1 | 1 | 0 | 1 | 0 | 0 | 1 | 1 | 1 | 1 | 1 | 7/10 |
| Yong-Wook 2015 | 1 | 0 | 0 | 1 | 0 | 0 | 0 | 1 | 1 | 1 | 1 | 5/10 |
| Yang 2020 | 1 | 1 | 1 | 1 | 0 | 0 | 0 | 1 | 1 | 1 | 1 | 7/10 |
| Peurala 2005 | 1 | 1 | 1 | 1 | 0 | 0 | 0 | 1 | 1 | 1 | 1 | 7/10 |
| Pandian 2014 | 1 | 1 | 1 | 1 | 1 | 0 | 1 | 1 | 1 | 1 | 1 | 9/10 |
| Korkusuz 2025 | 1 | 1 | 0 | 1 | 0 | 0 | 1 | 1 | 1 | 1 | 1 | 7/10 |
| Hung 2014 | 1 | 1 | 1 | 1 | 0 | 0 | 1 | 1 | 0 | 1 | 1 | 7/10 |
| Dias 2007 | 1 | 1 | 1 | 1 | 0 | 0 | 1 | 1 | 0 | 1 | 1 | 7/10 |
| Wu 2014 | 1 | 1 | 0 | 1 | 0 | 0 | 0 | 1 | 0 | 1 | 1 | 5/10 |
| Zhu 2016 | 1 | 1 | 1 | 1 | 0 | 0 | 1 | 1 | 1 | 1 | 1 | 8/10 |
| Immink 2014 | 1 | 1 | 1 | 1 | 0 | 0 | 1 | 1 | 0 | 1 | 1 | 7/10 |
| Hong 2020 | 1 | 1 | 0 | 1 | 0 | 0 | 0 | 0 | 0 | 1 | 1 | 4/10 |
| Kwag 2025 | 1 | 1 | 0 | 1 | 0 | 0 | 1 | 1 | 1 | 1 | 1 | 7/10 |
| Lee 2016 | 1 | 1 | 0 | 1 | 0 | 0 | 0 | 1 | 0 | 1 | 1 | 5/10 |
| Park 2021 | 1 | 1 | 1 | 1 | 1 | 0 | 1 | 1 | 0 | 1 | 1 | 8/10 |
| Bayouk 2006 | 1 | 1 | 0 | 1 | 0 | 0 | 0 | 1 | 0 | 1 | 1 | 5/10 |
| Bonan 2004 | 1 | 1 | 1 | 1 | 0 | 0 | 1 | 1 | 0 | 1 | 1 | 7/10 |
| Kim 2015 | - | - | - | - | - | - | - | - | - | - | - | 4/10 |
| Bang 2016 | - | - | - | - | - | - | - | - | - | - | - | 7/10 |
| Junata 2021 | - | - | - | - | - | - | - | - | - | - | - | 6/10 |
| Chang 2021 | - | - | - | - | - | - | - | - | - | - | - | 6/10 |
| Mustafaoğlu 2018 | - | - | - | - | - | - | - | - | - | - | - | 7/10 |
| Cui 2024 | - | - | - | - | - | - | - | - | - | - | - | 5/10 |
| Farqalit 2013 | - | - | - | - | - | - | - | - | - | - | - | 8/10 |
| Marigold 2005 | - | - | - | - | - | - | - | - | - | - | - | 6/10 |
| Chen 2014 | - | - | - | - | - | - | - | - | - | - | - | 7/10 |
| Globas 2012 | - | - | - | - | - | - | - | - | - | - | - | 7/10 |
| Middleton 2014 | - | - | - | - | - | - | - | - | - | - | - | 6/10 |
| Page 2008 | - | - | - | - | - | - | - | - | - | - | - | 4/10 |
| Wang 2015 | - | - | - | - | - | - | - | - | - | - | - | 6/10 |
| Yen 2008 | - | - | - | - | - | - | - | - | - | - | - | 7/10 |
| Chu 2004 | - | - | - | - | - | - | - | - | - | - | - | 6/10 |
| Jin 2012 | - | - | - | - | - | - | - | - | - | - | - | 4/10 |
| Pang 2005 | - | - | - | - | - | - | - | - | - | - | - | 8/10 |
| Quaney 2009 | - | - | - | - | - | - | - | - | - | - | - | 6/10 |
| Henrique 2019 | - | - | - | - | - | - | - | - | - | - | - | 5/10 |
| Park 2018 | - | - | - | - | - | - | - | - | - | - | - | 5/10 |
| Cho 2012 | - | - | - | - | - | - | - | - | - | - | - | 5/10 |
| Gok 2008 | - | - | - | - | - | - | - | - | - | - | - | 6/10 |
| Kim 2009 | - | - | - | - | - | - | - | - | - | - | - | 6/10 |
| Lee 2014 | - | - | - | - | - | - | - | - | - | - | - | 7/10 |
| Lee 2012 | - | - | - | - | - | - | - | - | - | - | - | 8/10 |
| Llorens 2015 | - | - | - | - | - | - | - | - | - | - | - | 8/10 |
| Cho 2014 | - | - | - | - | - | - | - | - | - | - | - | 7/10 |
| Kang 2012 | - | - | - | - | - | - | - | - | - | - | - | 7/10 |
| Westlake 2009 | - | - | - | - | - | - | - | - | - | - | - | 6/10 |
| Yang 2011 | - | - | - | - | - | - | - | - | - | - | - | 4/10 |
| Lau 2012 | - | - | - | - | - | - | - | - | - | - | - | 8/10 |
| Marin 2013 | - | - | - | - | - | - | - | - | - | - | - | 8/10 |
| Tankisheva 2014 | - | - | - | - | - | - | - | - | - | - | - | 7/10 |
| Rydwik 2006 | - | - | - | - | - | - | - | - | - | - | - | 5/10 |
| Saleh 2019 | - | - | - | - | - | - | - | - | - | - | - | 6/10 |
| Yu 2020 | - | - | - | - | - | - | - | - | - | - | - | 8/10 |
| Çoban 2024 | - | - | - | - | - | - | - | - | - | - | - | 5/10 |
| Yuen 2021 | - | - | - | - | - | - | - | - | - | - | - | 7/10 |
| Au-Yeung 2009 | - | - | - | - | - | - | - | - | - | - | - | 6/10 |
| Noh 2008 | - | - | - | - | - | - | - | - | - | - | - | 4/10 |
| Schmid 2012 | - | - | - | - | - | - | - | - | - | - | - | 6/10 |
| Suh 2014 | - | - | - | - | - | - | - | - | - | - | - | 6/10 |
| Lee 2018 | - | - | - | - | - | - | - | - | - | - | - | 4/10 |
| Choi 2016 | - | - | - | - | - | - | - | - | - | - | - | 6/10 |
| Zhao 2022 | - | - | - | - | - | - | - | - | - | - | - | 6/10 |
| Lee 2015 | - | - | - | - | - | - | - | - | - | - | - | 5/10 |
| Kim 2013 | - | - | - | - | - | - | - | - | - | - | - | 6/10 |
| Cho 2013 | - | - | - | - | - | - | - | - | - | - | - | 6/10 |
| Cha 2014 | - | - | - | - | - | - | - | - | - | - | - | 7/10 |
| Choi 2015 | - | - | - | - | - | - | - | - | - | - | - | 7/10 |
| Brogardh 2012 | - | - | - | - | - | - | - | - | - | - | - | 9/10 |
| Hyung Young Lee 2015 | - | - | - | - | - | - | - | - | - | - | - | 5/10 |

Note: a. Eligibility criteria were specified.
Description: The study clearly described the inclusion and/or exclusion criteria for participants.Scoring: Not included in the total score.

b. Subjects were randomly allocated to groups.
Description: Participants were assigned to groups using a randomization method (e.g., random number table, computer-generated sequence).Scoring: 1 point.

c. Allocation was concealed.
Description: The allocation sequence was hidden from investigators and participants until assignment (e.g., sealed envelopes, independent randomization center).Scoring: 1 point.

d. The groups were similar at baseline regarding the most important prognostic indicators.
Description: Groups did not differ significantly at baseline in key characteristics such as age, sex, disease severity, or duration.Scoring: 1 point.

e. There was blinding of all subjects.
Description: Participants were unaware of the group to which they were assigned.Scoring: 1 point.

f. There was blinding of all therapists who administered the therapy.
Description: The therapists delivering the intervention were blinded to participant group allocation.Scoring: 1 point.

g. There was blinding of all assessors who measured at least one key outcome.
Description: The outcome assessors were blinded to group allocation.Scoring: 1 point.

h. Measures of at least one key outcome were obtained from more than 85% of the subjects initially allocated to groups.
Description: At least 85% of participants completed follow-up assessments for key outcomes.Scoring: 1 point.

i. All subjects for whom outcome measures were available received the treatment or control condition as allocated, or data were analyzed by intention to treat.
Description: All randomized participants were analyzed in the groups to which they were assigned (intention-to-treat analysis).Scoring: 1 point.

j. The results of between-group statistical comparisons are reported for at least one key outcome.
Description: The study presented statistical comparisons (e.g., p-values, confidence intervals) between groups for at least one key outcome.Scoring: 1 point.

k. The study provides both point estimates and measures of variability for at least one key outcome.
Description: The study reported the mean ± SD, standard error, or confidence intervals for at least one primary outcome.Scoring: 1 point.

Total = 11 items (a–k)

Item a is not counted in the total score

Items b–k = 10 possible points

**Supplementary Table S5. GRADE certainty assessment for pooled outcomes**

| **Outcome** | **No. of studies** | **Study design** | **Risk of bias** | **Inconsistency** | **Indirectness** | **Imprecision** | **Other considerations / Notes** | **Intervention** | **Control** | **Relative effect** | **Absolute effect** | **Certainty** |
| --- | --- | --- | --- | --- | --- | --- | --- | --- | --- | --- | --- | --- |
| BBS | 43 | Randomized trials | Not serious | Not serious | Not serious | Not serious | Small-study effects detected; downgraded for publication bias. | 757 | 703 | - | MD 2.54 points higher (1.83 to 3.26 higher) | ⊕⊕⊕○ Moderate |
| TUG | 31 | Randomized trials | Not serious | Not serious | Not serious | Not serious | No serious publication bias detected. | 582 | 501 | - | MD 2.68 s lower (3.72 to 1.65 lower) | ⊕⊕⊕⊕ High |
| FRT | 10 | Randomized trials | Not serious | Serious | Not serious | Not serious | Moderate heterogeneity; downgraded for inconsistency. | 164 | 131 | - | SMD 0.65 higher (0.25 to 1.05 higher) | ⊕⊕⊕○ Moderate |

CI, confidence interval; MD, mean difference; SMD, standardized mean difference.

All included studies were randomized controlled trials (RCTs). According to the Cochrane Handbook and the GRADE Working Group guidance, evidence derived from RCTs starts as high certainty and may be downgraded based on five domains: risk of bias, inconsistency, indirectness, imprecision, and publication bias.

In this review, no serious concerns were identified for risk of bias, indirectness, or imprecision. Potential small-study effects were detected for the BBS outcome, and the certainty of evidence was therefore downgraded for publication bias. For the TUG outcome, no serious concerns were identified across the GRADE domains. For the FRT outcome, the certainty of evidence was downgraded for inconsistency because of moderate heterogeneity. Therefore, the certainty of evidence was rated as moderate for BBS, high for TUG, and moderate for FRT.

**Supplementary Table S6. Subgroup analyses of the effects of exercise-based interventions on balance and functional mobility in patients with chronic stroke.**

| Outcome | Subgroup | No. of Studies | Effect estimate  (MD, 95% CI) | Tests for heterogeneity | | p | Test for subgroup difference |
| --- | --- | --- | --- | --- | --- | --- | --- |
|  |  |  |  | I^2^ | τ^2^ |  |  |
| BBS | Balance and/or functional weight-shifting training | 10 | 3.70 (1.77, 5.63) | 63% | 5.69 | 0.0002 | Chi² = 37.56, df = 4, P < 0.00001, I² = 89.3% |
|  | Gait training | 14 | 2.92 (2.22, 3.61) | 0% | 0.00 | <0.00001 |  |
|  | Multisensory training | 8 | 0.51 (-0.57, 1.59) | 0% | 0.00 | 0.36 |  |
|  | High-intensity aerobic training | 4 | 0.32 (-0.69, 1.33) | 0% | 0.00 | 0.53 |  |
|  | Other | 7 | 4.63 (3.01, 6.25) | 0% | 0.00 | <0.00001 |  |
| TUG | Balance and/or functional weight-shifting training | 9 | -1.51 (-3.91, 0.88) | 63% | 6.93 | 0.22 | Chi² = 2.08, df = 3, P = 0.56, I² = 0% |
|  | Gait training | 6 | -3.16 (-5.57, -0.76) | 60% | 4.33 | 0.01 |  |
|  | Multisensory training | 6 | -2.54 (-3.76, -1.31) | 0% | 0.00 | < 0.0001 |  |
|  | Other | 10 | -3.59 (-5.40, -1.78) | 55% | 3.91 | 0.0001 |  |

**Supplementary Table S7. Exploratory meta-regression analyses of intervention duration and training frequency for BBS, TUG, and FRT outcomes**

| **Outcome / analysis** | **k** | **Predictor** | **β estimate** | **95% CI** | **P value** | **R²** |
| --- | --- | --- | --- | --- | --- | --- |
| BBS | 43 | Intervention duration (weeks) | −0.02 | −0.24 to 0.20 | 0.840 | 0% |
| TUG | 31 | Intervention duration (weeks) | 0.10 | −0.30 to 0.50 | 0.625 | 0% |
| FRT | 10 | Intervention duration (weeks) | −0.08 | −0.27 to 0.12 | 0.404 | 0% |
| BBS sensitivity analysis  (excluding condensed 10-day schedule) | 42 | Intervention duration (weeks) | −0.02 | −0.24 to 0.21 | 0.895 | 0% |
| BBS | 42 | Training frequency (sessions/week) | 0.35 | −0.31 to 1.01 | 0.292 | 0.65% |
| TUG | 30 | Training frequency (sessions/week) | 0.11 | −0.92 to 1.13 | 0.833 | 0% |
| FRT | 10 | Training frequency (sessions/week) | 0.18 | −0.27 to 0.63 | 0.385 | 0% |
| BBS sensitivity analysis  (excluding atypical daily home-practice schedule) | 41 | Training frequency (sessions/week) | 0.39 | −0.32 to 1.10 | 0.271 | 0.13% |

Abbreviations: BBS, Berg Balance Scale; TUG, Timed Up and Go; FRT, Functional Reach Test; CI, confidence interval. β represents the change in effect estimate associated with each additional week of intervention duration or each additional training session per week. Meta-regression models were estimated using restricted maximum likelihood with Knapp–Hartung adjustment.

**Supplementary Table S8. Sensitivity analyses using three-level random-effects meta-analysis accounting for country-level clustering**

| **Outcome** | **k** | **Original model** | **Three-level model** | **σ² Country** | **σ² Study** |
| --- | --- | --- | --- | --- | --- |
| BBS | 43 | MD 2.48 (0.99–3.97) | MD 2.52 (1.45–3.58) | 1.37 | 1.10 |
| TUG | 31 | MD −2.68 (−3.72–−1.65) | MD −2.69 (−3.75–−1.63) | 0 | 4.30 |
| FRT | 10 | SMD 0.65 (0.25–1.05) | **SMD 0.65 (0.25–1.06)** | 2.04 | 2.04 |

**Note:** Three-level random-effects meta-analyses were performed using restricted maximum likelihood estimation, with effect sizes nested within studies and studies nested within countries. σ² represents the estimated variance component attributable to country-level and study-level differences. MD, mean difference; SMD, standardized mean difference; CI, confidence interval.

**Supplementary Figure S1. Subgroup analysis of the effect of exercise-based interventions on Berg Balance Scale outcomes.**

**Supplementary Figure S2. Subgroup analysis of the effect of exercise-based interventions on Timed Up and Go outcomes.**

**Supplementary Figure S3. Funnel plot. A. BBS; B. TUG; C. FRT**

**Supplementary Figure S4. Egger’s test. A. BBS; B. TUG; C. FRT**

**Supplementary Figure S5. Post hoc subgroup analysis of the effect of exercise-based interventions on Berg Balance Scale outcomes according to comparator condition.**

**Supplementary Figure S6. Post hoc subgroup analysis of the effect of exercise-based interventions on Timed Up and Go outcomes according to comparator condition.**

**Supplementary Figure S7. Post hoc subgroup analysis of the effect of exercise-based interventions on Functional Reach Test outcomes according to comparator condition.**
